# Transforming Growth Factor-Beta-Regulated LncRNA-MUF Promotes Invasion by Modulating the miR-34a Snail1 Axis in Glioblastoma Multiforme

**DOI:** 10.3389/fonc.2021.788755

**Published:** 2022-02-08

**Authors:** Bakhya Shree, Shraddha Tripathi, Vivek Sharma

**Affiliations:** Department of Biological Sciences, Birla Institute of Technology and Science, Hyderabad, India

**Keywords:** glioblastoma, lncRNA, Snail1, miR-34a, TGF-β

## Abstract

Transforming growth factor beta (TGF-β)-regulated long-non-coding RNAs (lncRNAs) modulate several aspects of tumor development such as proliferation, invasion, metastasis, epithelial to mesenchymal transition (EMT), and drug resistance in various cancers, including Glioblastoma multiforme (GBM). We identified several novel differentially expressed lncRNAs upon TGF-β treatment in glioma cells using genome-wide microarray screening. We show that TGF-β induces lncRNA-MUF in glioma cells, and its expression is significantly upregulated in glioma tissues and is associated with poor overall survival of GBM patients. Knockdown of lncRNA-MUF reduces proliferation, migration, and invasion in glioma cells and sensitizes them to temozolomide (TMZ)-induced apoptosis. In addition, lncRNA-MUF downregulation impairs TGF-β-induced smad2/3 phosphorylation. In line with its role in regulating invasion, lncRNA-MUF functions as a competing endogenous RNA (ceRNA) for miR-34a and promotes Snail1 expression. Collectively, our findings suggest lncRNA-MUF as an attractive therapeutic target for GBM.

## 1 Introduction

GBM is a heterogeneous malignancy of the central nervous system characterized by aggressive invasion into the surrounding tissue (1). Despite following an aggressive treatment approach involving surgical resection and radio- and chemotherapy with temozolomide (TMZ), it remains incurable with a dismal survival rate of about 15 months (2). One of the characteristic features of GBM is extensive infiltration and invasion of the tumor cells to the surrounding parenchyma, which leads to colonization and relapse of tumors (3). TGF-β is a cytokine with multiple functions regulating cell proliferation, differentiation, and tissue homeostasis (4, 5). TGF-β promotes cancer cell invasion, EMT, and chemoresistance (5). TGF-β is overexpressed in glioblastoma, and its elevated expression is associated with the increased histologic grade of GBM (6). TGF-β promotes proliferation, invasion, metastasis, angiogenesis, resistance to apoptosis, replicative immortality, evasion of growth suppression, evasion of immune checkpoint blockade, and chemoresistance in GBM (7–9).

Less than 3% of the human genome codes for proteins, while the rest of the genome pervasively transcribes several non-coding transcripts (10–12). Among these, lncRNA transcripts are the most abundant and are loosely defined as longer than 200 bp in length with no ability to code for proteins (10, 13). LncRNAs interact with proteins or other non-coding RNAs to regulate gene expression in *cis* and *trans* to modulate cancer phenotypes (10, 14). Several differentially expressed lncRNAs have been identified in GBM, which regulate various aspects of GBM pathology (15–20). TGF-β-regulated lncRNAs modulate invasion, metastasis, and EMT in various cancers (21). In glioma, TGF-β-regulated lncRNAs lncRNA-ATB, UCA1, LINC00645, and LINC00115 modulate proliferation, invasion, and glioma stem cell renewal (22–25). In addition, TGF-β-induced lncRNAs H19 and HOXD-AS2 confer TMZ resistance in glioma by regulating miR-198 biogenesis and competing with KSRP (9).

Using a microarray screen, we identified several previously uncharacterized TGF-β1-regulated lncRNAs in T98G cells and characterized the role of one of the TGF-β-induced lncRNA-mesenchymal upregulated factor (lncRNA-MUF/LINC00941) in glioma physiology. LncRNA-MUF was first identified by Yan et al., and they demonstrated that it regulates EMT in hepatocellular carcinoma (HCC) (26). However, the functions and mechanism of action of lncRNA-MUF in GBM were not known. We show that levels of lncRNA-MUF are upregulated in GBM tumor samples along with histological grade. Our results suggest that it functions as an oncogenic lncRNA to promote glioma cell growth and invasion by functioning as a miRNA sponge for miR-34a that targets and suppresses Snail1. In addition, we show that lncRNA-MUF depletion sensitizes glioma cells to TMZ-induced apoptosis. Collectively, our results suggest that the lncRNA-MUF/miR-34a/Snail1 signaling axis may serve as a novel therapeutic target for GBM treatment.

## 2 Materials and Methods

### 2.1 Cell Culture and Treatments

T98G cells were purchased from the American Type Culture Collection (Manassas, VA). LN229, LN18, and U87-MG cells were purchased from NCCS, Pune. All cells were grown in complete medium, DMEM (Invitrogen) containing 10% fetal bovine serum (FBS) supplemented with 1 mM l-glutamine, and penicillin/streptomycin (Gibco). Cells were treated with TGF-β1 (10 ng/ml) PeproTech (#100-21) in serum-free medium (SFM) for dose and duration indicated in the figures and legends. SB505124 (Tocris # 3263), an inhibitor of TGFβRI/smad2/3, was used at a concentration of 6 µM for pretreatment of GBM cells to inhibit TGF-β signaling wherever indicated.

### 2.2 Microarray Analysis

Agilent SurePrint G3 Gene Expression Microarrays for Human (v3) for lncRNAs, containing 30,606 lncRNAs and 37,756 RefSeq-coding transcripts, were used to interrogate lncRNA and mRNA changes in vehicle versus TGF-β1 (10 ng/ml)-treated T98G cells after 24 h. RNA was isolated using MN NucleoSpin RNA Plus isolation kit (Cat. No. 740984.5). 10 µg of purified RNA samples was treated with recombinant DNAse I (Invitrogen Thermo Scientific—Cat. No. EN0521) as per manufacturer’s instructions, and the RNA samples were column purified using the MN NucleoSpin column purification kit. Hybridization and analysis were performed at the Molecular Genomics Core at Genotypic Technology (Bangalore). Briefly, total RNA was end-labeled using Agilent Quick-Amp labeling Kit (p/n5190-0442) and hybridized to Agilent Human Gene Expression Microarray 8X60K. Fragmentation of labeled cRNA and hybridization were done using the Gene Expression Hybridization kit (Agilent Technologies, *In situ* Hybridization Kit, # 5190-0404). The hybridized slides were scanned using the Agilent Microarray Scanner (Agilent Technologies, Part Number G2600D). Data analysis was done by using GeneSpring GX software version 14.5. Gene expression in the test group (TGF-β) was compared with the control group (C) to identify differentially expressed genes (DEGs) upon TGF-β treatment. DEGs were selected based on log base 2 (fold ≥ 0.6) and log base 2 (fold ≤-0.6) with a statistical significance of p-value < 0.05.

### 2.3 Bioinformatic Analysis

LncRNA-MUF expression values and associated prognostic information from 693 glioma cases were obtained from the Chinese Glioma Genome Atlas (http://www.cgga.org.cn). These 693 samples comprised 505 WHO III and IV tumors and 188 WHO II tumors. The Kaplan–Meier estimation method was used for the overall survival analysis of patients based on lncRNA expression. miRNA targets of lncRNA-MUF were predicted by the RNAInter database (http://rnainter.org/). The interaction between lncRNA-MUF and miR-34a was confirmed by RNAhybrid (https://bibiserv.cebitec.uni-bielefeld.de/rnahybrid?id=rnahybrid_view_submission) and IntaRNA (https://www.rna-society.org/rnainter/IntaRNA.html). mRNA targets of miR-34a were identified using the miR-DB (http://mirdb.org/) and TargetScan (http://www.targetscan.org/vert_72/) databases. Kaplan–Meier survival analysis of miR-34a was performed by using data from the CGGA database.

### 2.4 siRNA Transfection

Transfections of siRNAs were performed using Lipofectamine^®^ RNAiMAX Transfection Reagent (Life Technologies-Invitrogen, Cat. No.: 13778-075) and Opti-MEM (Invitrogen, 31985062) as per manufacturer’s instructions. Glioma cells were transfected with 40 nM of siRNAs (Silencer Pre-Designed siRNA, Ambion, Thermo Fisher Scientific) targeting lncRNA-MUF. The siRNA duplexes used in this study are as follows: si-MUF-1 sense 5′ GCCUUCAACAUUCAGCACATT 3′, antisense 5′ UGUGCUGAAUGUUCAAGGCTG 3′; si-MUF-2 sense 5′ CCUCCAUAUUCAUGAACUATT 3′, antisense 5′ UAGUUCAUGAAUAUGGAGGCT 3′. Non-specific siRNA that does not target any known mammalian gene was purchased from Dharmacon ON-TARGETplus non-targeting control pool (Cat. No.: D-001810-10-20). To overexpress or inhibit miR-34a, we transfected glioma cells with mimics of human miR-34a (80 nM) (miRCURY LNA miRNA mimic, Qiagen, Cat. No.: 339173) and with inhibitors of miR-34a (80 nM) (miRCURY LNA miRNA inhibitor, Qiagen, Cat. No.: 339121) using Lipofectamine according to the manufacturer’s instructions.

### 2.5 RNA Isolation and Real-Time PCR

Total RNA was extracted from glioma cells using the MN NucleoSpin RNA plus isolation kit (Cat. No.: 740984.5). 1 µg of RNA was converted into cDNA using the PrimeScript first-strand cDNA kit from Takara (#6110A). Quantitative real-time PCR (qRT-PCR) was performed with the SYBR Green PCR Kit (#RR820A, Takara) in the Bio-Rad CFX96 real-time qPCR system. All reactions were performed in triplicates and normalized with TBP/HPRT as an internal control. The relative gene expression of each sample was calculated using the 2^-ddct^ formula. For miRNA expression analysis, RNA was isolated using Zymo Quick-RNA™ Miniprep Plus Kit (#R1057). miRNA cDNA was synthesized using the mir-X miRNA 1^st^-Strand Synthesis Kit (#638313, Takara). qRT-PCR of miRNA was carried out using the universal primer and the primer specific to miR-34a-5p (**Supplementary Table 1**). U6 was used as a normalizing control. The gene specific primer sequences are shown in **Supplementary Table 1**.

### 2.6 Nuclear and Cytoplasmic Extract Preparations

Cytoplasmic and nuclear fractions were separated, and RNA was purified as described previously (27). qRT-PCR was performed to identify relative RNA levels in each fraction by using GAPDH as a control for cytoplasmic fraction, and MALAT1 as a control for nuclear fraction.

### 2.7 Western Blot Analysis

Whole-cell lysates were isolated from T98G and U87-MG glioma cells with lysis buffer containing Triton X (1%), NaCl (150 mM), Tris base (10 mM), EDTA (1 mM), EGTA (0.2 mM), IGEPAL (0.5%), protease inhibitor (3 µl/ml), and phosphatase inhibitors NaOVa_3_ (0.2 M) and NaF (0.5 M) 48 h after transfection. Cell lysates were incubated on ice for 20–30 min, with intermittent vortexing, and centrifuged at 14,000 rpm at 4°C for 20 min. The supernatant was collected in fresh prechilled tubes, and total protein was estimated using the BCA method, and extracts were frozen at −80°C until use. Western blotting was performed as described previously (28). Briefly, equal amounts of each sample protein were separated by sodium dodecyl sulfate polyacrylamide gel electrophoresis and transferred to a PVDF membrane, followed by blocking with 5% bovine serum albumin (A7906, Sigma) for 1.5 h. After that, the membrane was incubated with respective antibodies overnight at 4°C. The following primary antibodies (1: 2,500) were used: p-SMAD2/3 (CST #8828), total SMAD (CST #8685), Vimentin (CST #5741), N-cadherin (CST #13116), and Snail1 (CST #3895). Secondary antibodies (1:20,000)—HRP conjugated anti-rabbit (Vector Laboratories Cat. No.: P.I. 2000-1) or anti-mouse IgG (Invitrogen, Cat. No.: A16072)—were incubated for 2 h at room temperature. Immune blot bands were visualized with an ECL solution [Amersham ECL Prime Western Blotting Detection Reagent, GE Healthcare (Cat. No.: RPN2232)] and detected using VILBER Fusion Pulse ChemiDoc. Images were captured using Evolution Capture software. The blots were stripped and reprobed with β-actin antibody (1:100,000; Sigma #A1978) to determine equivalent loading as described previously (28). For stripping, blots were incubated at 50°C in stripping buffer containing 10% SDS, 0.5 M Tris–HCl, and 100 mM β-mercaptoethanol for 30 min, followed by PBST washes (5 times), blocking, and incubation with the primary antibody as described previously (29). The blot signals were quantified using ImageJ software for Microsoft Windows (National Institutes of Health, Bethesda, MD).

### 2.8 Chromatin Immunoprecipitation (ChIP)

Control or TGF-β-treated cells were fixed with 1% formaldehyde for 8 min at room temperature followed by quenching with a final concentration of 0.125 M glycine for 5 min. Cells were washed twice with ice-cold phosphate-buffered saline, harvested by scraping, pelleted, and resuspended in 250 μl of ChIP lysis buffer (50 mM Tris–HCl [pH 8.1], 0.9% SDS, 10 mM EDTA, protease inhibitor), and samples were incubated on ice for 1 h. Samples were sonicated using Bioruptor Plus (Diagenode) with sonication conditions: 30 s on, 30 s off for 20 cycles. After sonication, samples were centrifuged at 14,000 rpm at 4°C for 20 min. Supernatants were diluted 5-fold in ChIP dilution buffer (167 mM NaCl, 16.7 mM Tris–HCl pH 8.1, 1.2 mM EDTA, 1.1% Triton X-100, protease inhibitors), and 5% of the sample was taken as input. Samples were then incubated at 4°C overnight with Smad2/3 (1:200) (CST #8685S) or anti-rabbit IgG (CST #2729S) antibody. The following day, Dynabeads and protein G (Invitrogen #10004D) 50 μl per sample were added to the I.P. tubes and incubated for 2 h at 4°C. The beads were then washed once each with low-salt buffer (0.1% SDS, 1% Triton X-100, 2 mM EDTA, 20 mM Tris–HCl pH 8.1, 150 mM NaCl), high-salt buffer (0.1% SDS, 1% Triton X-100, 2 mM EDTA, 20 mM Tris HCl pH 8.1, 500 mM NaCl), LiCl buffer (0.25 M LiCl, 1% NP-40, 1% deoxycholate, 1 mM EDTA, 10 mM Tris–HCl pH 8.1), and TE buffer (pH 8.0). Following this, samples were reverse cross-linked by using decrosslinking buffer (222 mM NaCl, 50 mM Tris, 10 mM EDTA, 0.025% SDS), containing 5 μl proteinase K (NEB #P8107S, 800 U/ml) per sample with overnight incubation at 65°C. Genomic DNA was then extracted with a DNA purification kit (Zymo Kit Cat. No.: D3020), and lncRNA-MUF in immunoprecipitated samples was measured using qRT-PCR. The following primers specific to the lncRNA-MUF promoter were used for qRT-PCR analysis: forward primer, 5′ CTCAGTGCCTTCATGGTGGA 3′ reverse primer: 5′ GAGGGGCTTACAGATGTGGC 3′.

### 2.9 Cell Proliferation Assay

Colorimetric cell proliferation assay was performed by using the WST-1 reagent (Cat#. 05015944001, Roche) at the indicated time according to the manufacturer’s instructions. Briefly, cells were seeded at a concentration of 2,500–5,000 cells/well in 96-well plates and transfected with siRNAs si-MUF-1, si-MUF-2, and si-NS at 40 nM, and cell proliferation was quantified at OD of 450 nm.

### 2.10 Colony Formation Assay

For clonogenic assays, cells were seeded into 96-well dishes and treated with si-NS or siRNAs against lncRNA-MUF. 24 h post-transfection, cells were trypsinized and seeded at a density of 200 cells per well in a 6-well dish and incubated at 37°C. Media were changed every 3 days. Colonies formed 14 days after plating were fixed with 4% paraformaldehyde and stained with crystal violet solution, and counted.

### 2.11 Caspase 3/7 Assay

Luminometric assay kit for caspase-3/7 (Promega, G8090) was used to determine the enzymatic activity of caspase-3/7 in glioma cells transfected with si-MUF-1 and 2. 48 h post-transfection, proluciferin DEVD substrate and caspase-Glo 3/7 buffer were added to the cells, and the assay was performed as per the manufacturer’s instructions.

### 2.12 Invasion Assay

Glioma cells were transfected with control siRNA (si-NS) or si-MUF-1/si-MUF 2. After 24 h of transfection, cells were seeded into the upper chamber of transwell inserts (Corning, #3422) precoated with Matrigel (Corning, 356234) in serum-free media. Lower chambers had media containing 20% FBS. After 48 h, cells remaining on the upper surface of the membrane were gently removed with a cotton swab. Invaded cells were fixed with 4% PFA and stained with crystal violet solution (Sigma, V5265). Stained cells were visualized under Magnus INVI microscopy (×100), and invaded cells were counted at four different fields for each condition (23).

### 2.13 Migration Assays

For migration assay, si-MUF-1/si-MUF-2 or negative control siRNA-transfected glioma cells were seeded in a 12-well dish and cultured overnight. Scratch was made using a 20-µl pipette tip followed by PBS wash. Cells were maintained in 0.5% serum-containing media. Images of scratch were taken at 0, 24, and 48 h, and the migrating length was calculated using ImageJ (30).

### 2.14 Dual-Luciferase Reporter Assay

Dual-luciferase reporter assays were done to confirm the interaction between miR-34a-5p and lncRNA-MUF. The lncRNA-MUF region with miR-34a-5p sites was cloned into the pmirGLO vector (Promega) using NheI and SalI restriction sites. Cells were co-transfected with pmirGLO-lncRNA-MUF reporter plasmid and miR-34a-5p/N.C. mimics using Polyplus jetPRIME transfection reagent. 30 h post-transfection, the cells were lysed and subjected to luciferase assays using the Dual-Luciferase^®^ Reporter Assay System (Promega, Cat. No.: E1910) according to the manufacturer’s instructions on the SpectraMax iD3 Luminometer (Molecular Devices Corporation). Data were normalized to Renilla luciferase activity (23, 26).

For lncRNA-MUF promoter analysis, we cloned the -734-bp promoter region of lncRNA-MUF with predicted putative SBEs into the restriction sites of SacI and NheI of pGL3basic luciferase and renilla_polyA construct (a gift from Oskar Laur) (Addgene plasmid # 128046; http://n2t.net/addgene:128046; RRID:Addgene_128046). T98G and U87-MG cells were seeded at ~60%–70% confluency in 24-well plates. The next day, they were transiently transfected with 0.3 μg of lncRNA-MUF promoter containing pGL3basic luciferase and renilla_polyA reporter plasmid using jet prime transfection reagent. Eighteen hours post-transfection, cells were serum-starved for 6 h followed by treatment with 10 ng/ml TGF-β1 for the indicated time. Luciferase activity was measured using the Dual-Luciferase^®^ Reporter Assay System according to the manufacturer’s protocol (Promega) on the SpectraMax iD3 Luminometer (Molecular Devices Corporation). The results are expressed as a fold change in luciferase activity over control (28).

### 2.15 Statistical Analysis

Results are presented as mean ± SEM unless otherwise stated. We used paired Student’s t-test for comparisons between two experimental groups. Additional statistical tests information is described in the figure legends. p < 0.05 was considered statistically significant.

## 3 Results

### 3.1 Identification of TGF-β-Regulated LncRNAs in GBM Cells Using Microarray Screen

**Figure 1:**
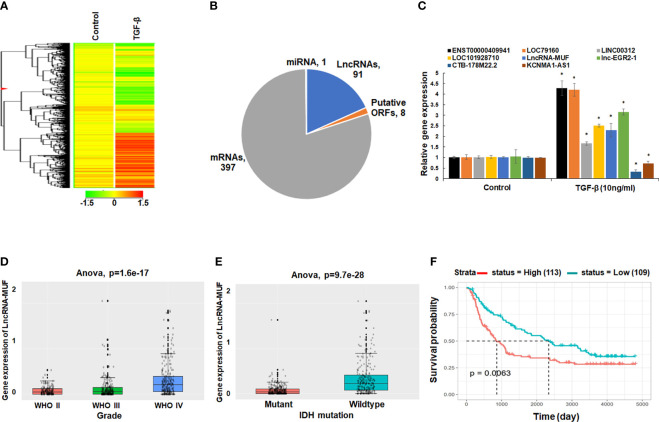
LncRNA-MUF is overexpressed in GBM, and it is induced by TGF-β. **(A)** Heatmap representing the relative abundance of differentially expressed genes (DEGs, ≥1.5-fold, p < 0.05) in T98G cells upon TGF-β1 (10 ng/ml) treatment for 24 h. **(B)** Pie chart representing the class of DEGs (p < 0.05) identified from genome-wide microarray screening in T98G GBM cells upon TGF-β1 treatment for 24 h. **(C)** Validation of top TGF-β-regulated lncRNAs identified from microarray screening using qRT-PCR in T98G cells. RNA samples were analyzed by qRT-PCR, and error bars represent the mean ± SEM from three independent experiments. *Significant change in TGF-β-treated cells compared to control cells (p < 0.05). **(D)** LncRNA-MUF expression analysis in glioma tissues from the CGGA database. Elevated expression of MUF in GBM IV compared to lower-grade gliomas (p=1.6e-^17^). **(E)** LncRNA-MUF levels are elevated in IDH wild-type GBM patients compared to IDH mutant cases as analyzed from the CGGA database (ANOVA, p = 9.7e-^28^). **(F)** Kaplan–Meier survival analysis of lncRNA-MUF expression in glioma samples from CGGA databases shows that a high lncRNA-MUF expression is associated with poor overall survival in primary and recurrent GBM patients (p = 0.0063). Red line represents high lncRNA-MUF expression group, and green line represents the low lncRNA-MUF expression group.

We sought to identify, in an unbiased fashion and at a genome-wide scale, differentially expressed lncRNAs upon TGF-β treatment in glioma cells. We performed gene expression analysis of control, and TGF-β1-treated T98G glioma cells using the Agilent SurePrint G3 Gene Expression Microarrays for Human (v3) for lncRNAs. Using a 1.5-fold change and p-value < 0.05 as a threshold, we identified 91 differentially expressed lncRNAs and 397 differentially expressed mRNAs in our screen (**Figures 1A, B** and **Supplementary Table 2**). LncRNAs constitute 18.3% of transcripts among the total number of DEGs identified upon TGF-β1 treatment in T98G cells (**Figure 1B**). We verified the TGF-β1-induced gene expression changes in levels of lncRNAs in T98G cells using qRT-PCR (**Figure 1C**). LncRNAs ENST00000409910 and LOC79160 get ~4-fold upregulated upon TGF-β treatment (**Figure 1C**). LncRNAs LINC00312, LOC101928710, lncRNA-MUF, and lnc-EGR2-1 get ~1.5–3-fold upregulated upon TGF-β treatment (**Figure 1C**). LncRNAs CTB-178M22.2 and KCNMA1-AS1 are significantly downregulated upon TGF-β treatment (**Figure 1C**). The expression of several TGF-β-regulated mRNAs identified from the microarray screen was also verified using qRT-PCR (**Figure S1A**). Among these upregulated lncRNAs, we further set out to characterize the role of lncRNA-MUF in glioma pathogenesis.

### 3.2 LncRNA-MUF Is Upregulated in GBM Tumor Samples and Is Associated With Poor Patient Prognosis

To investigate the role of lncRNA-MUF in GBM pathophysiology, we decided to evaluate its expression in GBM tumor samples using the CGGA database (http://www.cgga.org.cn/). Using the mRNAseq_693 dataset of the CGGA database, we found that levels of lncRNA-MUF were significantly higher in GBM samples than normal brain tissues (p = 6.3e-^15^) (**Figure S1B**). Moreover, lncRNA-MUF levels are significantly higher in grade IV GBM than in lower-grade gliomas (p = 1.6e-^17^) (**Figure 1D**). GBM patients with IDH mutation show a better survival rate than the IDH wild-type group (1). Hence, we evaluated the expression of lncRNA-MUF in IDH mutant and wild-type glioma samples. We observed that the expression of lncRNA-MUF is significantly higher in gliomas with the IDH wild-type group than in the IDH mutant group (p = 9.7e-^28^) (**Figure 1E**). In addition, high expression of lncRNA-MUF is correlated with poor overall survival in both primary and recurrent GBM patients (p = 0.0063) (**Figure 1F**). These results suggest that lncRNA-MUF expression is associated with aggressive phenotype and poor survival in glioma patients.

### 3.3 LncRNA-MUF Is Induced by TGF-β Through the Canonical SMAD2/3 Signaling Pathway in Glioma Cell Lines

**Figure 2:**
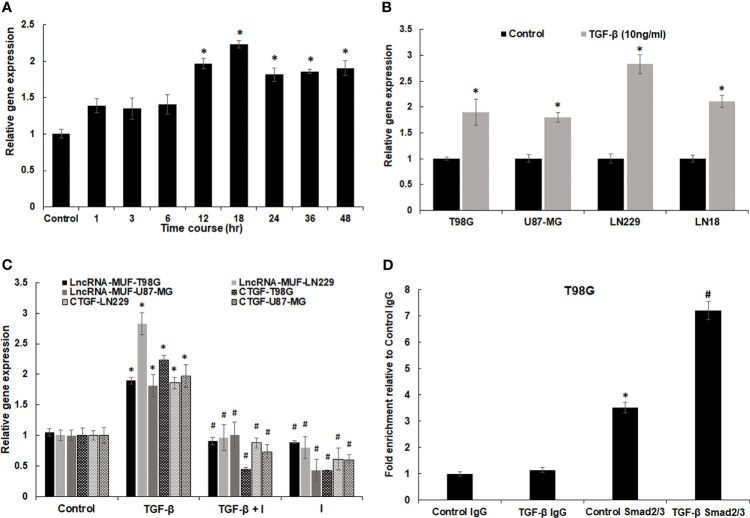
Regulation of lncRNA-MUF expression through Smad2/3 signaling. **(A)** LncRNA-MUF is a delayed transcript with induction of ~2-fold at 12 and 18 h of TGF-β1 treatment (10 ng/ml), then reaching a plateau of ~1.8-fold at 24, 36, and 48 h. RNA levels were measured at the indicated time points using qRT-PCR. **(B)** LncRNA-MUF induction upon TGF-β treatment is not cell type-specific. The indicated GBM cell lines were treated with 10 ng/ml TGF-β for 24 h and lncRNA-MUF levels measured by qRT-PCR. **(C)** LncRNA-MUF induction upon TGF-β treatment is smad2/3 dependent. Human glioma cells (T98G, LN229, and U87-MG) were pretreated with 6 µM of SB505124 (TGFβR1/Smad2/3 inhibitor) for 2 h followed by co-treatment with TGF-β1 (10 ng/ml) for 24 h, and lncRNA-MUF transcript levels were determined by qRT-PCR. **(D)** ChIP-qPCR analysis of smad2/3 interaction with SBE in the lncRNA-MUF promoter in control and TGF-β-treated T98G cells. DNA was isolated from control and TGF-β-treated cells after immunoprecipitation with the anti-smad2/3 antibody and was amplified using specific primer sets. LncRNA-MUF promoter levels in immunoprecipitated samples were measured by qRT-PCR analysis, normalized to input, and represented as “fold enrichment relative to control IgG I.P.” Values represent mean ± S.D. from two independent experiments. *Significant change compared to IgG (p < 0.05). #Significant change compared to control Smad2/3 (p < 0.05). Data information: RNA samples were analyzed by quantitative RT-PCR, normalized with TBP/HPRT. Error bars represent the mean ± SEM from 3 independent experiments. *Significant change compared to respective control samples (p < 0.05). ^#^Significant decrease from TGF-β-treated cells (p < 0.05). Statistical comparisons were made using Student’s t-test.

LncRNA-MUF induction upon TGF-β1 treatment was dose-independent for TGF-β doses from 5 to 80 ng/ml for 24 h in T98G cells (**Figure S2A**). The time-course analysis-identified lncRNA-MUF gets induced upon TGF-β1 treatment as early as 1 h. However, a statistically significant increase of ~2-fold occurs only at 12 and 18 h of TGF-β treatment and then sustained at ~1.8-fold at 24, 36, and 48 h (**Figure 2A**). To assess the impact of TGF-β1 on the lncRNA-MUF expression on additional glioblastoma cell lines, we evaluated the lncRNA-MUF expression in LN18, LN229, U87-MG glioma cells. Upon TGF-β1 stimulation for 24 h, the expression of lncRNA-MUF was upregulated (≥2-fold) in glioma cell lines (T98G: 1.89-fold; U87-MG: 1.8-fold; LN229: 2.8-fold; LN18: 2.1-fold). These results indicate that lncRNA-MUF induction upon TGF-β1 treatment is not cell line-specific (**Figure 2B**). We then investigated the subcellular localization of lncRNA-MUF by measuring the lncRNA levels in nuclear and cytoplasmic fractions in T98G and U87-MG GBM cells. LncRNA-MUF showed 75% expression in the cytoplasm and 25% in the nucleus in T98G and U87-MG cell lines, respectively (**Figure S2B**).

TGF-β signal transduction occurs either through the canonical smad2/3 signaling pathway or through non-canonical pathways (4). To identify the transcription factors working downstream of the TGF-β pathway to regulate lncRNA-MUF expression, we looked at the lncRNA-MUF promoter using JASPAR (http://jaspar.genereg.net/) and found functional smad-binding elements (SBE) (5′ CAGAC 3′/5′ GTCTG 3′) at the -498, -1,321, -1,850, -2,413, and -2,942 positions. Hence, we evaluated lncRNA-MUF expression upon TGF-β treatment in the presence and absence of TGF-β inhibitor SB505124. To this end, GBM cells were treated with 6 µm SB505124 (TGFβR1/smad2/3 inhibitor) for 2 h before treatment with TGF-β1 (24 h). Blocking smad2/3 with SB505124 significantly abrogated TGF-β-induced lncRNA-MUF expression in glioma cells (~50% reduction in T98G, LN229, and U87-MG) (**Figure 2C**). As TGF-β-induced lncRNA-MUF expression and TGF-βRI inhibitor significantly abrogated lncRNA-MUF levels, we used luciferase reporter assay to confirm if lncRNA-MUF promoter can drive TGF-β-mediated luciferase activity. Transfection of T98G and U87-MG cells with the lncRNA-MUF-promoter-luciferase reporter construct followed by TGF-β treatment for 24 h resulted in a significant ~2.5- and 2-fold increase in luciferase activity over control, respectively (**Figure S2C**). Next, we performed ChIP-qPCR to determine whether TGF-β promotes increased binding of smad2/3 to SBE on the lncRNA-MUF promoter. ChIP-qPCR revealed increased binding of smad2/3 on SBE on the lncRNA-MUF promoter upon TGF-β stimulation (**Figure 2D**). These results suggest that TGF-β upregulates lncRNA-MUF expression through the canonical SMAD signaling pathway.

### 3.4 Knockdown of LncRNA-MUF Reduces Cell Proliferation, Induces Apoptosis, and Sensitizes Glioma Cells to TMZ-Mediated Apoptosis

**Figure 3:**
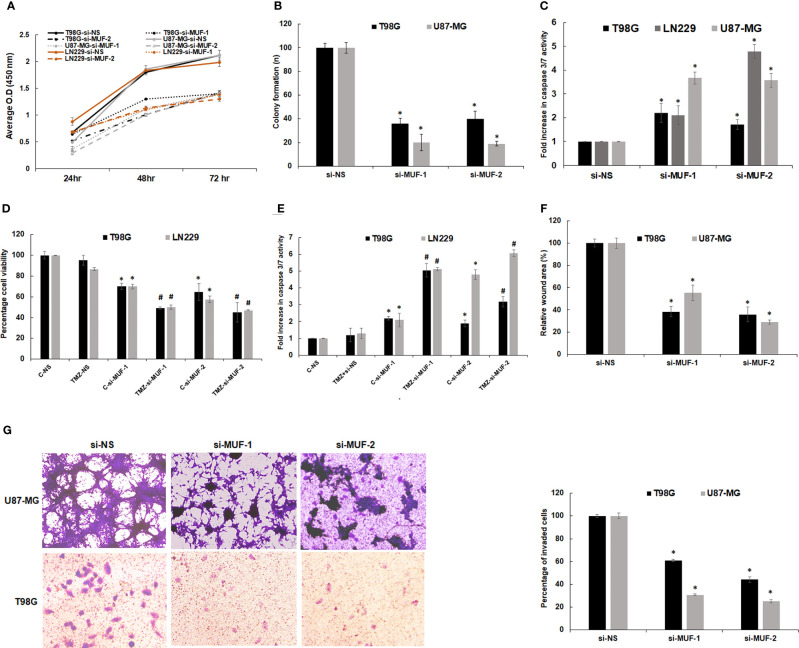
Effect of lncRNA-MUF knockdown on glioma cell proliferation, migration, and invasion *in vitro*. **(A)** Human glioma cells were transfected with si-NS or si-MUF-1/si-MUF-2 (40 nM), and percentage cell viability was calculated at indicated times using WST1. Values represent mean ± S.D. from four independent experiments. *Significant change compared to si-NS cells at the corresponding time (p < 0.05). Statistical comparisons were made using Student’s t-test. **(B)** Reduced colony formation ability of GBM cells with lncRNA-MUF knockdown. **(C)** Caspase 3/7 activity assay shows that lncRNA-MUF knockdown induces apoptosis in GBM cells. **(D)** LncRNA-MUF knockdown with a low dose of si-MUF1/si-MUF-2 (20 nM) in combination with TMZ treatment (600 µM) shows enhanced reduction of glioma cell (T98G and LN229) proliferation and increased sensitivity to TMZ, as analyzed using WST1 assay. **(E)** LncRNA-MUF knockdown (20 nM of si-MUF-1/si-MUF-2) combined with TMZ treatment (600 µM) show enhanced caspase 3/7 activity as compared to TMZ alone. **(F)** Wound healing assay demonstrates reduced GBM cell migration upon lncRNA-MUF knockdown. **(G)** Matrigel invasion assay shows that lncRNA MUF inhibition reduces glioma cell invasion. Values represent mean ± S.D. from four independent experiments. *Significant change compared to si-NS cells at the corresponding time (p < 0.05). Statistical comparisons were made using Student’s t-test. # Significant decrease from C-si-MUF-1 and C-si-MUF-2 trabsfected cells (p < 0.05).

To investigate the physiological function of lncRNA-MUF in glioma pathogenesis, we established lncRNA-MUF knockdown by siRNA using two different siRNAs (si-MUF-1 and si-MUF-2) in T98G and U87-MG cell lines. The knockdown of lncRNA-MUF with si-MUF-1 results in ~85% reduction, and si-MUF-2 results in ~67% reduction of lncRNA-MUF levels in T98G and U87-MG cells (**Figure S3**). LncRNA-MUF depletion using siRNAs results in a time-dependent reduction in cell proliferation in glioma cells. Cell proliferation was reduced by ~40% and ~55% at 48 and 72 h post-lncRNA-MUF knockdown, respectively, in T98G cells (**Figure 3A**). A similar ~40%–50% reduction in cell proliferation was observed in LN229 and U87-MG glioma cells transfected with siRNA against lncRNA-MUF compared to cells transfected with non-specific siRNA (si-NS) (**Figure 3A**). Consistent with the reduction in cell proliferation upon lncRNA-MUF depletion, MUF knockdown resulted in a significant decrease in colony formation of ~62% and 70%, respectively, in T98G and U87-MG cells compared to respective control cells transfected with si-NS (**Figure 3B** and **Figure S4C**). Moreover, depletion of lncRNA-MUF by siRNA also results in apoptosis as demonstrated by an increase of ~1.75-fold, 3.6-fold, and 3.4-fold caspase 3/7 activity in T98G, U87-MG, and LN229, respectively, as compared to control cells (**Figure 3C**). Consistently, the levels of caspase 9 mRNA were ~2-fold increased following lncRNA-MUF knockdown in T98G and U87-MG cells (**Figure S4A**). TMZ is an oral alkylating drug that is used to treat GBM; however, 50% of GBM cases develop resistance to TMZ. Several GBM cell lines such as T98G and LN229 show resistance to TMZ, and TGF-β-induced lncRNAs are known to promote TMZ resistance (9, 31). Therefore, we evaluated the effect of lncRNA-MUF knockdown on TMZ sensitivity in T98G and LN229 cells. LncRNA-MUF depletion with low siRNA levels (20 nM) resulted in significantly reduced cell proliferation in TMZ-treated T98G and LN229 cells compared to si-NS-transfected cells treated with TMZ (**Figure 3D**). In addition, TMZ treatment in lncRNA-MUF knockdown resulted in a significantly higher increase in caspase 3/7 activity (~5-fold) compared to si-NS-transfected T98G and LN229 cells treated with TMZ (**Figure 3E**). These results suggest that lncRNA-MUF knockdown sensitizes glioma cells to TMZ-induced apoptosis. We then investigated the effect of lncRNA-MUF knockdown on glioma cell migration and invasion. Wound healing assay revealed that lncRNA-MUF-depleted T98G and U87-MG cells show ~63% and ~58% reduction in cell migration, respectively, compared to control cells (**Figures 3F** and **S4B**). Matrigel invasion assay during lncRNA-MUF depletion results in ~55% and ~70% inhibition of cell invasion in T98G and U87-MG cells, respectively, compared to control cells (**Figure 3G**). Thus, collectively these results suggest that lncRNA-MUF serves as an oncogene to promote proliferation, drug resistance, migration, and invasion in GBM cells, and targeting lncRNA-MUF is an attractive therapeutic strategy for GBM.

### 3.5 LncRNA-MUF Regulates Gene Expression of a Subset of TGF-β Target Genes in cis and trans

**Figure 4:**
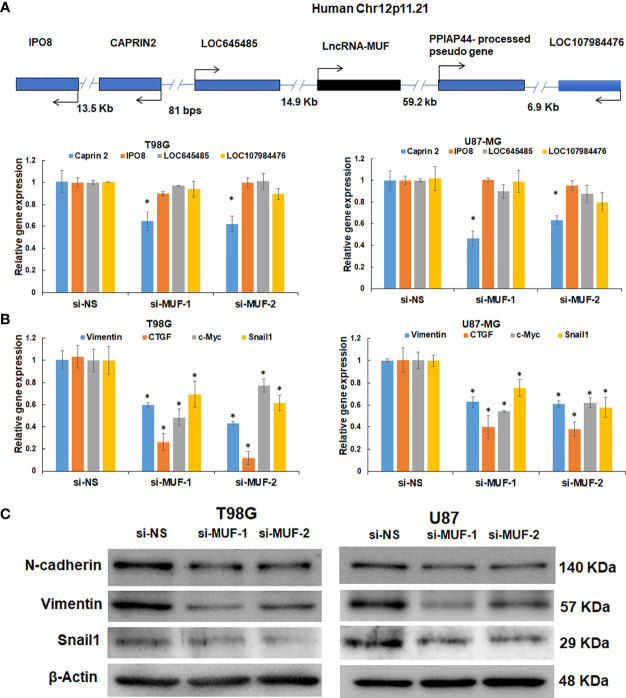
LncRNA-MUF modulates gene expression in cis and trans and promotes EMT in glioma. **(A)** Validation of cis gene expression of lncRNA-MUF with lncRNA knockdown. LncRNA knockdown represses cis gene expression (Caprin2). **(B)** LncRNA-MUF modulates TGF-β target gene expression in trans in GBM. T98G and U87-MG glioma cells transfected with si-NS, si-MUF-1, or si-MUF-2 (25 nM), and transcript levels of indicated genes were measured 48 h post-transfection. RNA samples were analyzed by quantitative RT-PCR, and error bars represent the mean ± SEM from three independent experiments. **(C)** LncRNA-MUF promotes GBM EMT. Western blot analysis of EMT markers Vimentin, N-cadherin, and Snail1 followed by lncRNA-MUF knockdown in T98G and U87-MG cells. *Significant change compared to cells transfected with si-NS (p < 0.05). Values represent mean ± SD from three independent experiments.

LncRNA transcripts often regulate gene expression in cis and trans (10). We first evaluated the effect of lncRNA-MUF knockdown on its cis genes (**Figure 4A**). We observed ~50% downregulation of the Caprin2 gene in T98G and U87-MG upon lncRNA-MUF knockdown with both the siRNAs (**Figure 4A**). This is consistent with Ai et al., who reported the cis-regulation of the Caprin2 gene by lncRNA-MUF through chromosome looping in OSCC (32). However, the levels of other cis genes (IPO8, LOC645485, LOC107984476) remained unchanged upon lncRNA-MUF knockdown (**Figure 4A**). We also observed that the Caprin2 gene is upregulated by TGF-β in T98G GBM cells (1.7-fold) using qPCR assays (**Figure S5A**). These results suggest that lncRNA-MUF regulates TGF-β-induced expression of the Caprin2 gene in *cis* in glioma cells. Since lncRNA-MUF regulates genes involved in the WNT/β-catenin pathway (26, 32), we evaluated the impact of lncRNA-MUF knockdown on the WNT/β-catenin pathway genes in glioma cells. Surprisingly, we did not observe any significant change in their expression upon MUF depletion in glioma cells (**Figure S5B**). To further identify the genes regulated in *trans* by lncRNA-MUF in glioma cells, we evaluated the expression of the TGF-β gene ontology group upon its siRNA-mediated knockdown. Depleting MUF resulted in ~50% downregulation of Snail1, ~40% downregulation of vimentin, ~60% downregulation of CTGF, and ~30% downregulation of c-Myc in T98G cells and U87-MG cells (**Figure 4B**). Several other TGF-β-regulated genes did not show any change in expression with MUF knockdown (**Figure S5C**). Since lncRNA-MUF depletion inhibits invasion and Snail1 regulates EMT and invasion, we evaluated EMT marker expression upon lncRNA-MUF inhibition by Western blotting. In agreement with q-PCR data, knockdown of lncRNA-MUF resulted in ~40% decrease in N-cadherin, ~80% decrease in vimentin, and ~70% decrease in Snail1 protein levels in T98G and U87-MG cells (**Figures 4C** and **S5D**). These results indicate that lncRNA-MUF selectively regulates the expression of Snail1, vimentin, N-cadherin, CTGF, and c-Myc in GBM.

### 3.6 Knockdown of LncRNA-MUF Attenuates TGF-β Signaling

**Figure 5:**
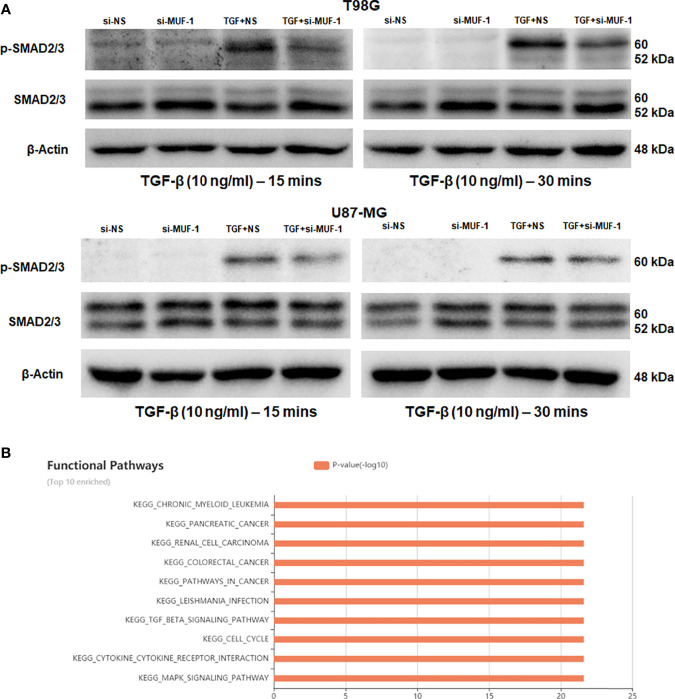
LncRNA-MUF regulates TGF-β signaling. **(A)** LncRNA-MUF knockdown impairs phosphorylation of the smad2/3 complex. Western blot analysis of psmad2/3 and total smad2/3 levels in T98G and U87-MG cells treated with 10 ng/ml TGF-β1 (15 and 30 min), 48 h after lncRNA-MUF knockdown with si-MUF-1. A representative blot is shown from three independent experiments with similar results. Blots were reprobed for β-actin to establish equivalent loading. **(B)** Top 10 enriched signaling pathways regulated by lncRNA-MUF identified from the lncACTdb database.

TGF-β-induced lncRNAs are known to regulate the TGF-β signaling pathway *via* an autocrine signaling loop (33). Hence, we asked if lncRNA-MUF is also involved in regulating TGF-β signaling. To test this, we evaluated the impact of lncRNA-MUF knockdown on TGF-β-induced phosphorylation of smad2/3. Silencing lncRNA-MUF in T98G results in a ~35% decrease in smad2/3 phosphorylation at 15 min and 30 min post-TGF-β treatment compared to si-NS cells treated with TGF-β. A similar reduction of ~30% is observed in p-smad2/3 levels upon TGF-β treatment in U87-MG cells compared to TGF-β1-treated si-NS cells (**Figures 5A** and **S6**). This is consistent with the fact that pathway analysis by the lncACTdb database suggests that the TGF-β signaling pathway is among the top 10 enriched signaling pathways regulated by lncRNA-MUF (**Figure 5B**). Our results indicate that lncRNA-MUF regulates smad2/3 phosphorylation downstream of the TGF-β pathway in glioma cells.

### 3.7 LncRNA-MUF Modulates TGF-β-Induced Invasion in Glioma *via* the miR-34a-5p/Snail1 Axis

**Figure 6:**
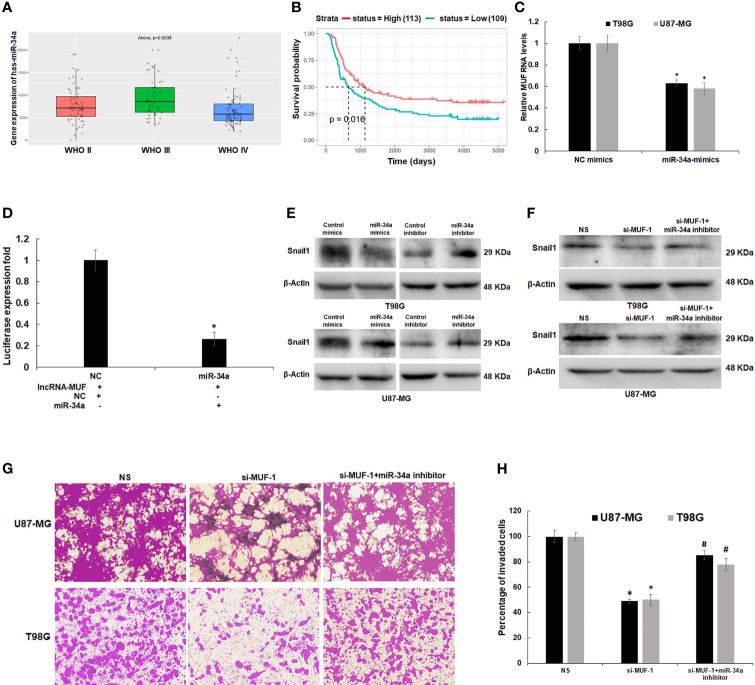
LncRNA-MUF acts as a ceRNA by sponging miR-34a and regulates Snail1 expression. **(A)** Low expression of miR-34a in grade IV GBM as compared to lower-grade gliomas (p = 0.0038). Red bar represents miR-34a expression in the WHO grade II group, green bar represents miR-34a expression in WHO grade III, and blue bar represents miR-34a expression in the WHO grade IV glioma group. **(B)** Kaplan–Meier survival analysis showed that a high miR-34a expression was correlated with better survival in primary and recurrent GBM patients identified from the CGGA database (p = 0.016). Red line represents the high miR-34a expression group, and green line represents the low miR-34a expression group. **(C)** Downregulation of lncRNA-MUF transcript levels upon miR-34a overexpression measured by qRT-PCR in T98G and U87-MG cells. **(D)** Luciferase activity assay demonstrated that lncRNA-MUF could bind with miR-34a; relative luciferase activity was measured in HEK293T cells co-transfected with miR-34a-5p mimics and pmiRGLO-lncRNA-MUF constructs. Luminescence signals were measured 30 h post-transfection using dual luciferase assay. Data are shown as mean ± S.D. of three independent experiments; *p < 0.05. **(E)** Downregulation and upregulation of Snail1 protein levels upon treatment with miR-34a mimics and miR-34a inhibitor, respectively. T98G and U87-MG cells transfected with 80 nM of negative control mimics/inhibitor and miR-34a mimics/inhibitor. 48 h post-transfection protein lysates were collected, and Snail1 protein levels were analyzed by Western blotting. A representative blot is shown from three independent experiments with similar results. Blots were reprobed for β-actin to establish equivalent loading. **(F)** Rescue of Snail1 protein levels caused by lncRNA-MUF knockdown in T98G and U87-MG glioma cells upon miR-34a inhibition. A representative blot is shown from three independent experiments with similar results. Blots were reprobed for β-actin to establish equivalent loading. **(G)** Rescue of invasion caused by lncRNA-MUF knockdown in T98G and U87-MG glioma cells upon miR-34a inhibition. **(H)** Quantification of invaded cells upon lncRNA-MUF knockdown and miR-34a inhibition in T98G and U87-MG cells. # Significant decrease from C-si-MUF-1 transfected cells (p < 0.05).

LncRNAs function as endogenous miRNA sponges and participate in the ceRNA regulatory network (34, 35). Yan et al. have reported the direct binding of lncRNA-MUF and miR-34a using RNA immunoprecipitation (RIP) and RNA pull-down assays (26). In addition, they show that lncRNA-MUF regulates Snail1 expression by sponging miR-34a to modulate EMT in HCC cells (26). Using RNAhybrid and IntaRNA databases, we identified putative miR-34a-binding sites in lncRNA-MUF (**Figure S7A**). To identify the interaction between lncRNA-MUF and miR-34a-5p, we cloned the region of lncRNA-MUF with the miR-34a-binding sites into the pmirGLO vector downstream of the firefly luciferase gene. Co-transfection with the pmirGLO-lncRNA-MUF reporter plasmid and miR-34a mimics reduced the reporter activity significantly (~70%) compared to the control cells (**Figure 6D**).

Since miR-34a has a well-established tumor-suppressor role in several cancers, including GBM (36), we first evaluated its expression in glioblastoma tissue using the CGGA dataset. Expression of miR-34a is lowest in grade IV GBM (p = 0.0038) (**Figure 6A**). In addition, the Kaplan–Meier survival curve demonstrates that high expression of miR-34a positively correlates with better survival of glioma patients (p = 0.016) (**Figure 6B**). To understand the impact of miR-34a on lncRNA-MUF regulation, we first determined its levels upon miR-34a overexpression using miRNA mimics. We observed a significant ~40%–50% reduction in lncRNA-MUF expression in T98G and U87-MG cells upon treatment with miR-34a mimic (**Figure 6C**). Snail1 is a well-known target of miR-34a; consistent with this, we observed that transfection of miR-34a mimics in T98G and U87-MG glioma cells significantly reduced Snail1 protein levels and knockdown of miR-34a using miRNA inhibitors reversed this effect (**Figure 6E** and **Figure S7B**). Given that miR-34a targets lncRNA-MUF and Snail1 expression and because we observed downregulation of Snail1 upon lncRNA-MUF depletion, we explored if lncRNA-MUF could act as a ceRNA to sponge miR-34a for stabilizing Snail1 to regulate invasion in glioma cells. Invasion analysis revealed that reduction in invasion upon lncRNA-MUF knockdown is significantly reversed upon co-transfection with the miR-34a inhibitor (**Figures 6G, H**). Moreover, in accordance with the role of miR-34a in the regulation of invasion by Snail1, we observed that the miR-34a inhibitor significantly restores Snail1 downregulation caused by lncRNA-MUF depletion (**Figure 6F** and **Figure S7C**). These experiments indicate that TGF-β induced lncRNA-MUF sponges miR-34a to promote Snail1-induced invasion (**Figures 6G, H**).

## 4 Discussion

Glioma is the most lethal and invasive malignant brain neoplasm with a poor prognosis and frequent recurrence after surgery. Prominent features of GBM that contribute to recurrence include the presence of glioma stem cells, resistance to TMZ, and invasion (25, 37). TGF-β secreted by glioma cells confers them with an aggressive pro-invasive phenotype and TMZ resistance (9). TGF-β induces the expression of several lncRNAs (LINC00645, LINC00115, UCA1, lnc-ATB) through the canonical or non-canonical signaling pathway to promote glioma progression (22–24, 30). Nie et al. identified eight differentially regulated lncRNAs (H19, HOXD-AS2, LINC00635, LINC00277, RP11-196G11.2, LINC00152, MALAT1, and LOC100506207) in D54, P-GBM2 cells (9). They demonstrated that H19 and HOXD-AS2 confer TMZ resistance by regulating miR-198 biogenesis by competing with KSRP (9). LINC00115 regulates glioma stem cell tumorigenicity by enhancing ZNF596 by preventing the binding of miR-200 to the 5′ UTR of ZNF596 (25).

We identify several novel differentially expressed lncRNAs upon TGF-β treatment using a genome-wide microarray screen in T98G cells. Among the identified lncRNAs, we unveil the role of lncRNA-MUF in glioma pathobiology. LncRNA-MUF, also known as mesenchymal stem cell upregulated factor (lncRNA-MUF), promotes hepatocellular carcinoma by binding to ANXA2 to activate WNT/β-catenin signaling-mediated EMT (26). In addition, it sponges miR-34a in HCC cells leading to upregulation of Snail1 to promote EMT (26). We demonstrate that the levels of lncRNA-MUF are elevated in GBM tumor samples, and its expression is associated with poor survival and prognosis. This is consistent with the fact that the levels of lncRNA-MUF are also upregulated in gastric cancer, oral squamous cell carcinoma (OSCC), papillary thyroid carcinoma, colorectal cancer (CRC), lung cancer, colon cancer, and pancreatic cancer (32, 38–46).

ChIP-seq revealed that the lncRNA-MUF promoter upon TGF-β stimulation accumulates, activating H3K27ac marks (38). LncRNA-MUF induction by TGF-β in CRC cells is abrogated upon treatment with disitertide, an inhibitor of TGFβR1 (39). In line with these findings, we show that lncRNA-MUF induction by TGF-β is completely abrogated upon treatment with TGFβR1/smad 2/3 inhibitor SB505124 in glioma cells (**Figure 2C**). TGF-β-regulated lncRNA-MIR100HG regulates smad2/3 phosphorylation in prostate carcinoma (33). LncRNA-MUF also regulates the TGF-β signaling by preventing the SMAD4 degradation by competing with β-TrCP in CRC (39). We demonstrate for the first time that MUF downregulation attenuates TGF-β-induced phosphorylation of smad 2/3 in glioma cells. LncRNA-MUF promotes OSCC progression by mediating chromosome looping to the promoter of its cis gene, Caprin2, to activate the WNT/β-catenin signaling-mediated progression of OSCC (32). Although we observed a significant downregulation of the Caprin2 gene with lncRNA-MUF knockdown, we did not observe any change in the WNT/β-catenin signaling genes. Our results suggest that apart from regulating Caprin2 expression, lncRNA-MUF modulates the expression of several genes involved in the TGF-β pathway in glioma cells (vimentin, CTGF, c-Myc, and Snail1) with Snail1 as one of the primary targets. However, the mechanism of regulation of vimentin, N-cadherin, CTGF, and c-Myc by lncRNA-MUF needs further investigation.

**Figure 7:**
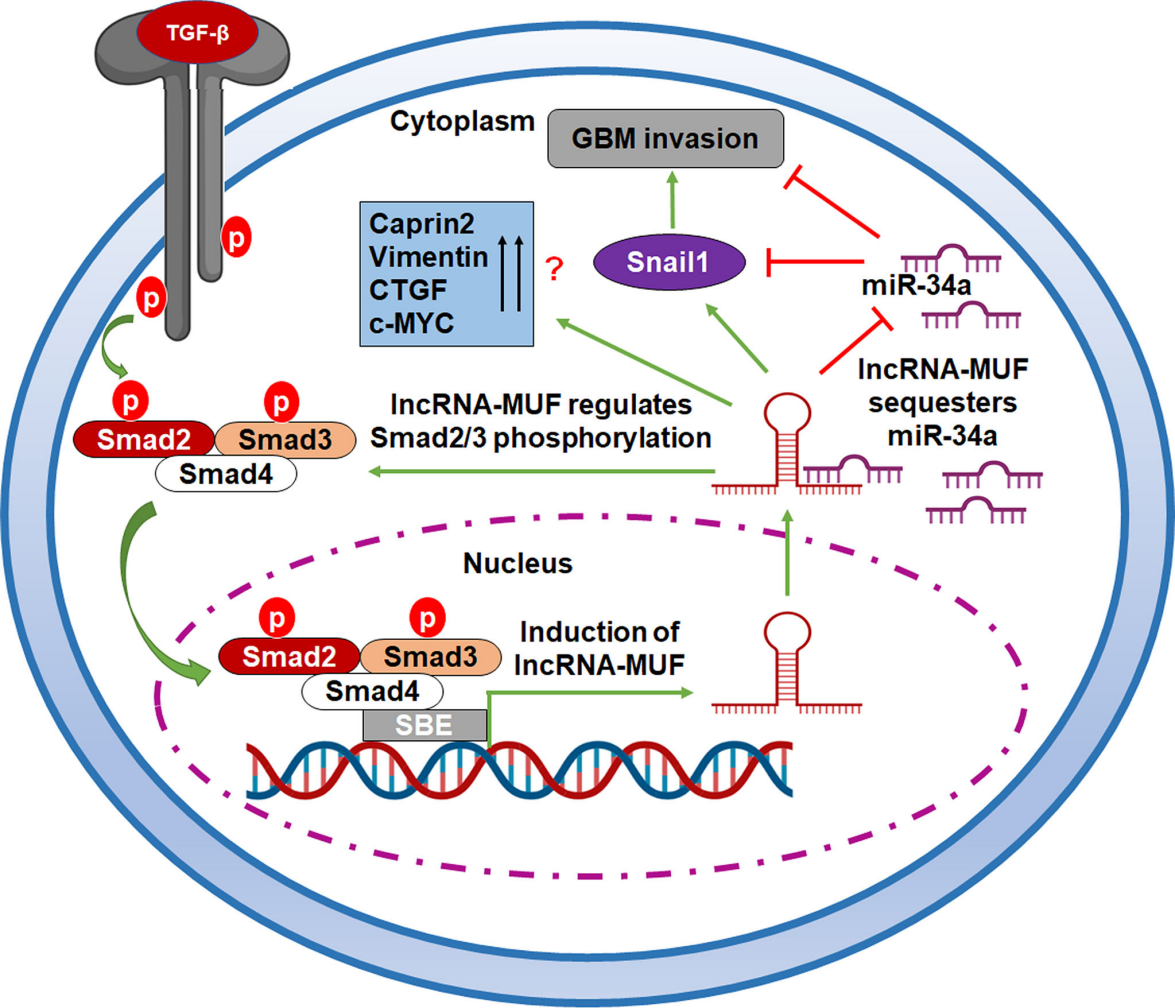
A working model of TGF-β-activated lncRNA-MUF-driven glioma invasion. LncRNA-MUF is activated through the canonical TGF-β pathway, and it in turn regulates the phosphorylation of Smad2/3. It sequesters miR-34a from its binding target, Snail1, resulting in enhanced Snail1 expression and glioma invasion through the lncRNA-MUF/miR-34a/Snail1 signaling axis.

LncRNAs act as endogenous miRNA sponges for binding to miRNAs or participating in the ceRNA regulatory network (35). The cross talk between miRNAs and TGF-β-induced lncRNAs regulates the EMT and tumor invasion in glioma (23, 25). miR-34a suppresses the proliferation and invasion in glioma (47). It is downregulated in human glioma tumors as compared to normal brain tissue (47). miR-34a has a potential tumor-suppressor role in glioma by targeting several oncogenes and also induces differentiation of glioma stem cells (47). Dai et al. recently reported that LINC00665 sponges miR-34a, which targets the angiotensin II receptor type I (AGTR1) gene to impede glioma malignancy (48). Several studies have reported that Snail1 is a direct target of miR-34a (36, 49, 50). Snail1 is a crucial transcription factor that promotes tumor cell invasion and EMT (51). Snail1 is often upregulated in glioma, and high expression of Snail1 is associated with poor survival of glioma patients (52). We observed a positive correlation between MUF and Snail1 expression in GBM tumor samples (**Figure S7D**). We also show that lncRNA-MUF depletion in glioma cells results in reduced migration and invasion, and lncRNA-MUF promotes GBM invasion by acting as an endogenous sponge for miR-34a and causing stabilization of its target Snail1 (**Figure 6G**). In addition, we show that loss of lncRNA-MUF expression reduces cell proliferation, induces apoptosis, and sensitizes glioma cells to TMZ-induced cell death. Our findings suggest that the TGF-β-regulated lncRNA-MUF/miR-34a/Snail1 signaling axis is a critical regulator of invasion in GBM (**Figure 7**). Our results warrant further preclinical studies on lncRNA-MUF using low-passage glioma patient-derived cell models, glioma stem cells, and *in vivo* models to firmly establish its role as a therapeutic target for GBM.

## Data Availability Statement

The datasets presented in this study can be found in online repositories. The names of the repository/repositories and accession number(s) can be found in the following: https://www.ncbi.nlm.nih.gov/, GSE183211.

## Author Contributions

BS: conceptualization, methodology, validation, formal analysis, investigation, visualization, writing—original draft. ST: methodology, validation, investigation, visualization. VS: conceptualization, methodology, investigation, visualization, formal analysis, resources, supervision, project administration, funding acquisition, writing—original draft, review, and editing. All authors contributed to the article and approved the submitted version.

## Funding

This work was supported by extramural grants from the Government of India (DST-SERB ECR/2017/001953 and DBT-RLS 102/IFD/SAN/3499/2016-17) and intramural funds from an OPERA grant from BITS Pilani to VS. BS is supported by an SRF from ICMR No. 2020-7940/GEN-BMS.

## Conflict of Interest

The authors declare that the research was conducted in the absence of any commercial or financial relationships that could be construed as a potential conflict of interest.

## Publisher’s Note

All claims expressed in this article are solely those of the authors and do not necessarily represent those of their affiliated organizations, or those of the publisher, the editors and the reviewers. Any product that may be evaluated in this article, or claim that may be made by its manufacturer, is not guaranteed or endorsed by the publisher.
