# Supplementary material for: Transforming Growth Factor-Beta-Regulated LncRNA-MUF Promotes Invasion by Modulating the miR-34a Snail1 Axis in Glioblastoma Multiforme
Source: Front Oncol. 2022 Feb 8;11:788755. doi: 10.3389/fonc.2021.788755 (PMC8865078; doi:10.3389/fonc.2021.788755)
Supplement: Supplementary file 1 [file DataSheet_1.docx]

**Supplementary figures and tables**

**Figure S1A. qRT-PCR validation of differentially expressed mRNAs upon TGF-β1 treatment** **identified from microarray screen.**

**
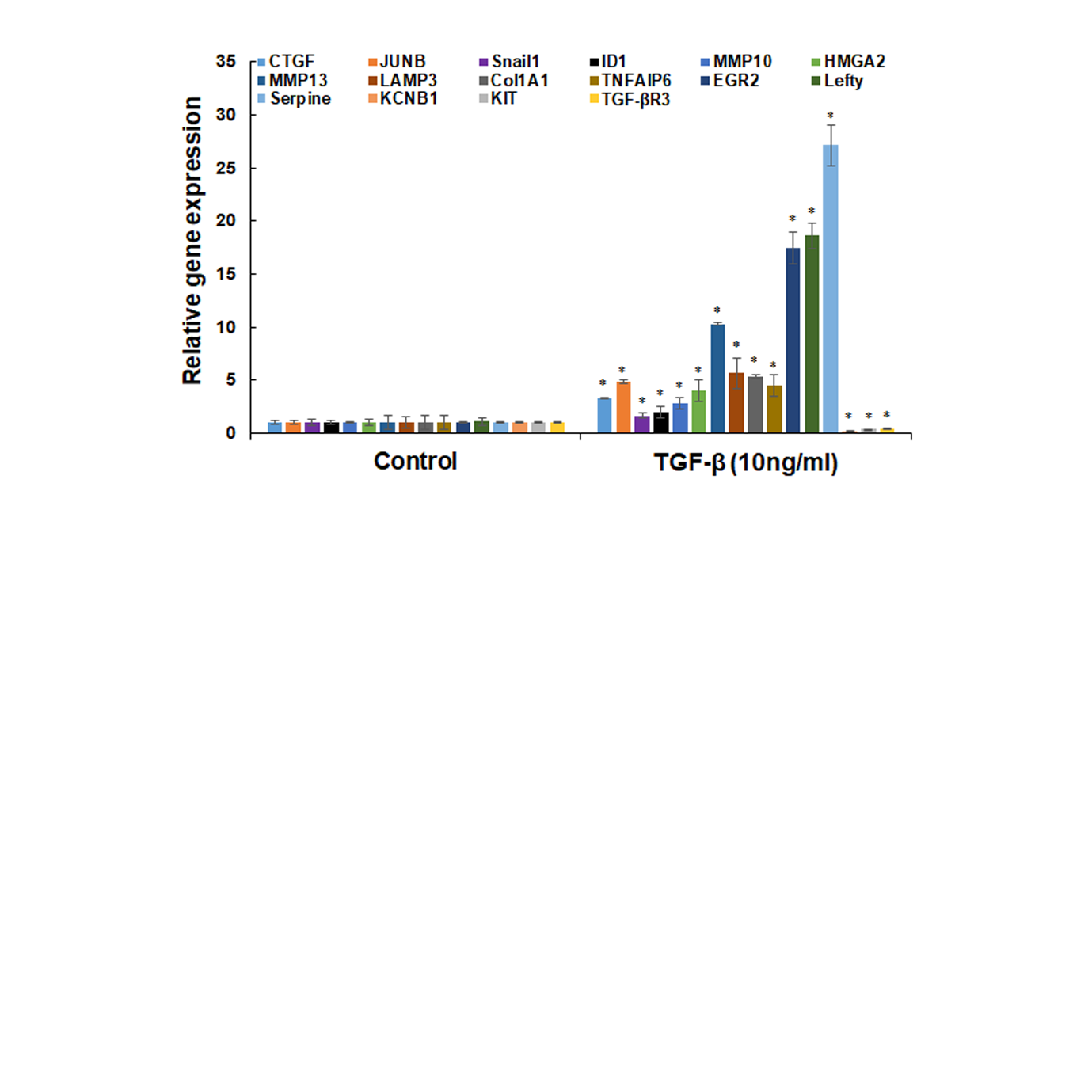
**

Human T98G glioma cells were treated with 10 ng/ml of TGF-β1 (24h) and transcript levels were measured using qRT-PCR, and error bars represent the mean ± SEM from three independent experiments. *Significant change compared to control cells (p < 0.05).

**Figure S1B. Levels of lncRNA MUF were significantly higher in GBM samples than normal brain tissues (p=6.3e-^15^) identified from CGGA database.**

**
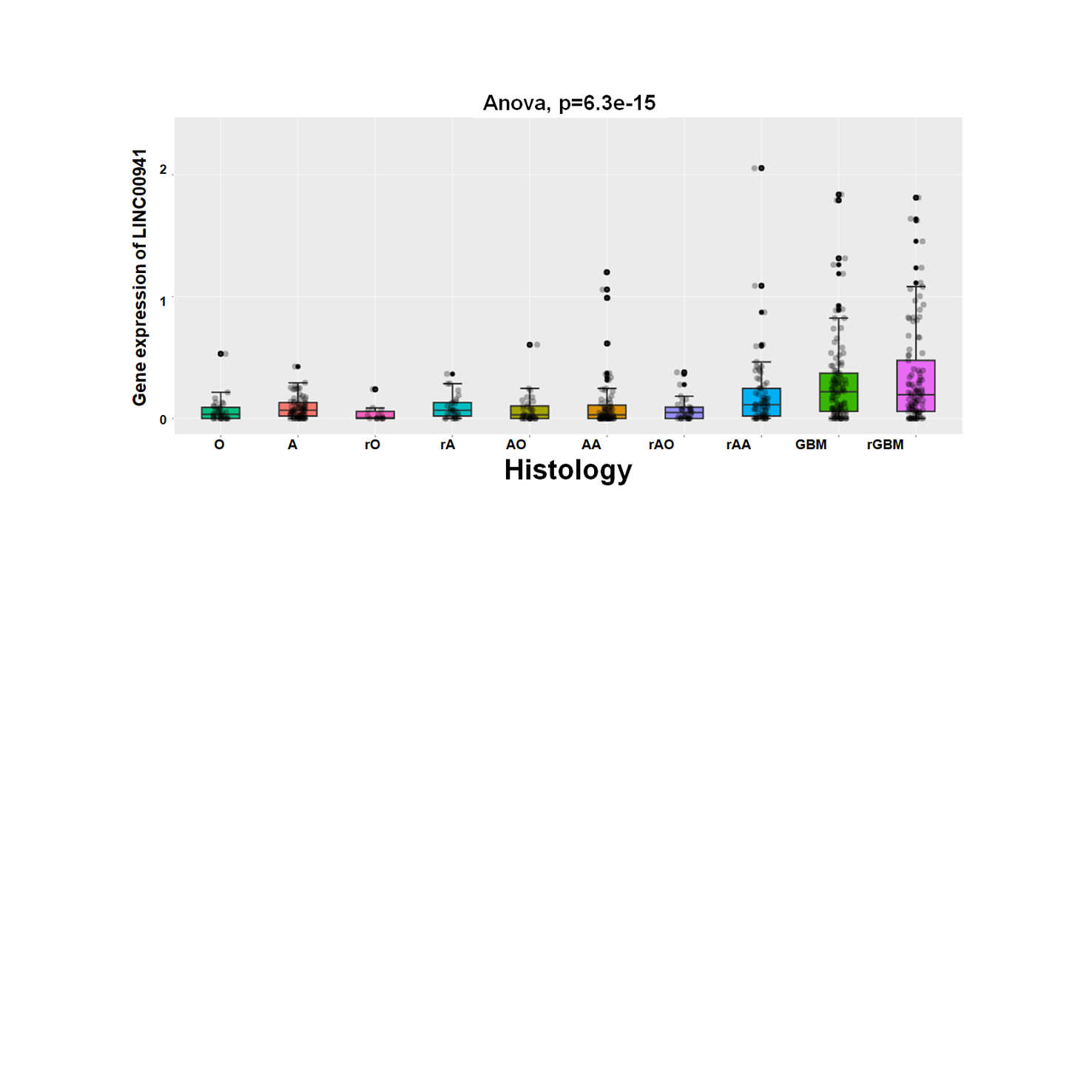
**

LncRNA-MUF levels were significantly higher in GBM samples than normal brain tissues (p=6.3e-^15^) identified from CGGA database; O – oligodendrocytoma; A – astrocytoma; rO – recurrent oligodendrocytoma; rA – recurrent astrocytoma; AO - anaplastic oligodendroglioma; AA - anaplastic astrocytoma; rAO – recurrent anaplastic oligodendroglioma; rAA – recurrent anaplastic astrocytoma; GBM – glioblastoma multiforme; rGBM – recurrent glioblastoma multiforme.

**Figure S2A. qRT-PCR of lncRNA-MUF with different doses of TGF-β1 treatment in T98G GBM cells**

**
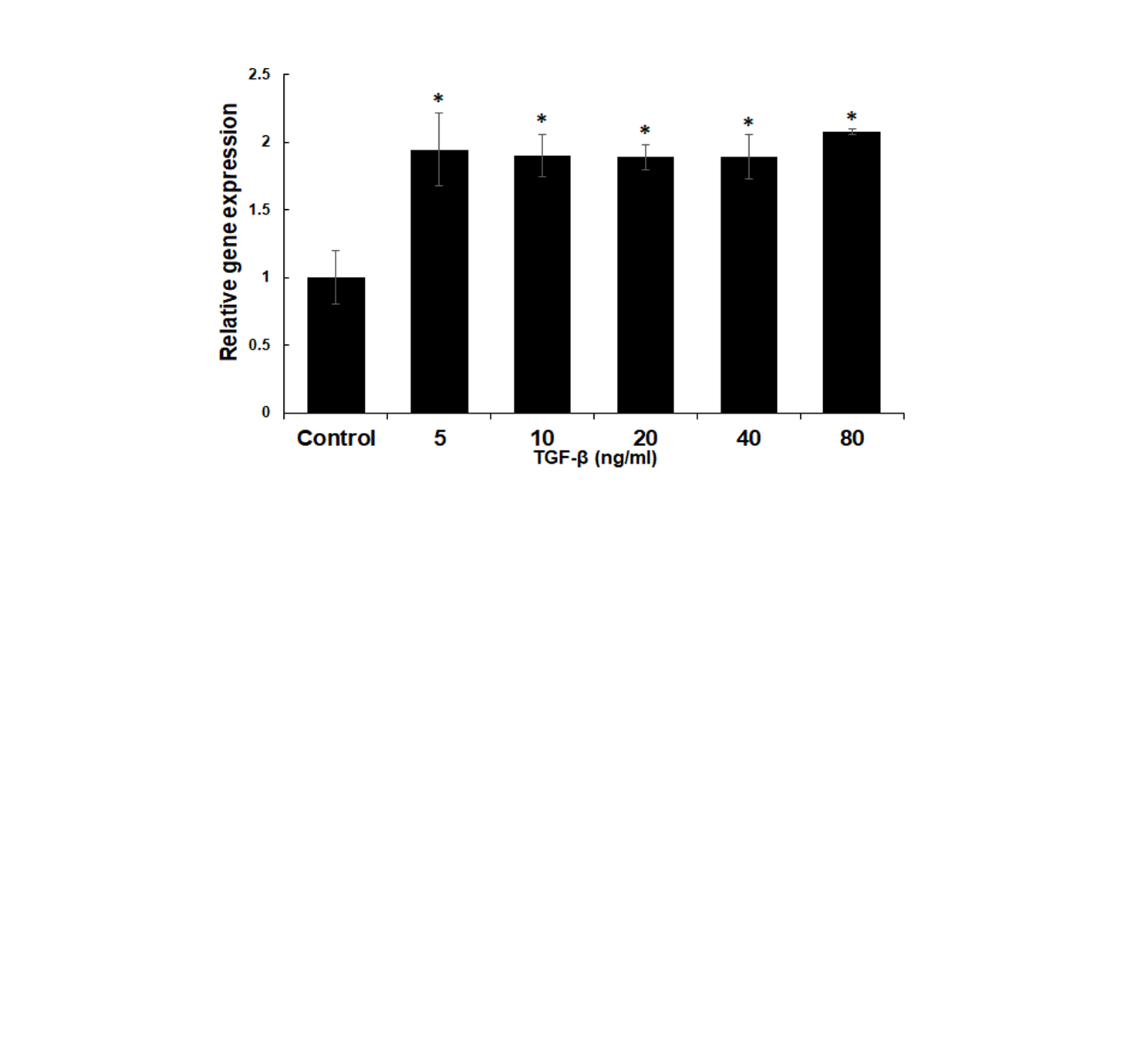
**

LncRNA-MUF induction upon TGF-β1 treatment is not dose dependent. Cells were treated with the indicated concentration of TGF-β1 for 24 h and analyzed for lncRNA-MUF expression by qRT-PCR.

**Figure S2B. Fractionation of GBM cells followed by qPCR to determine the localization of lncRNA–MUF.**

**
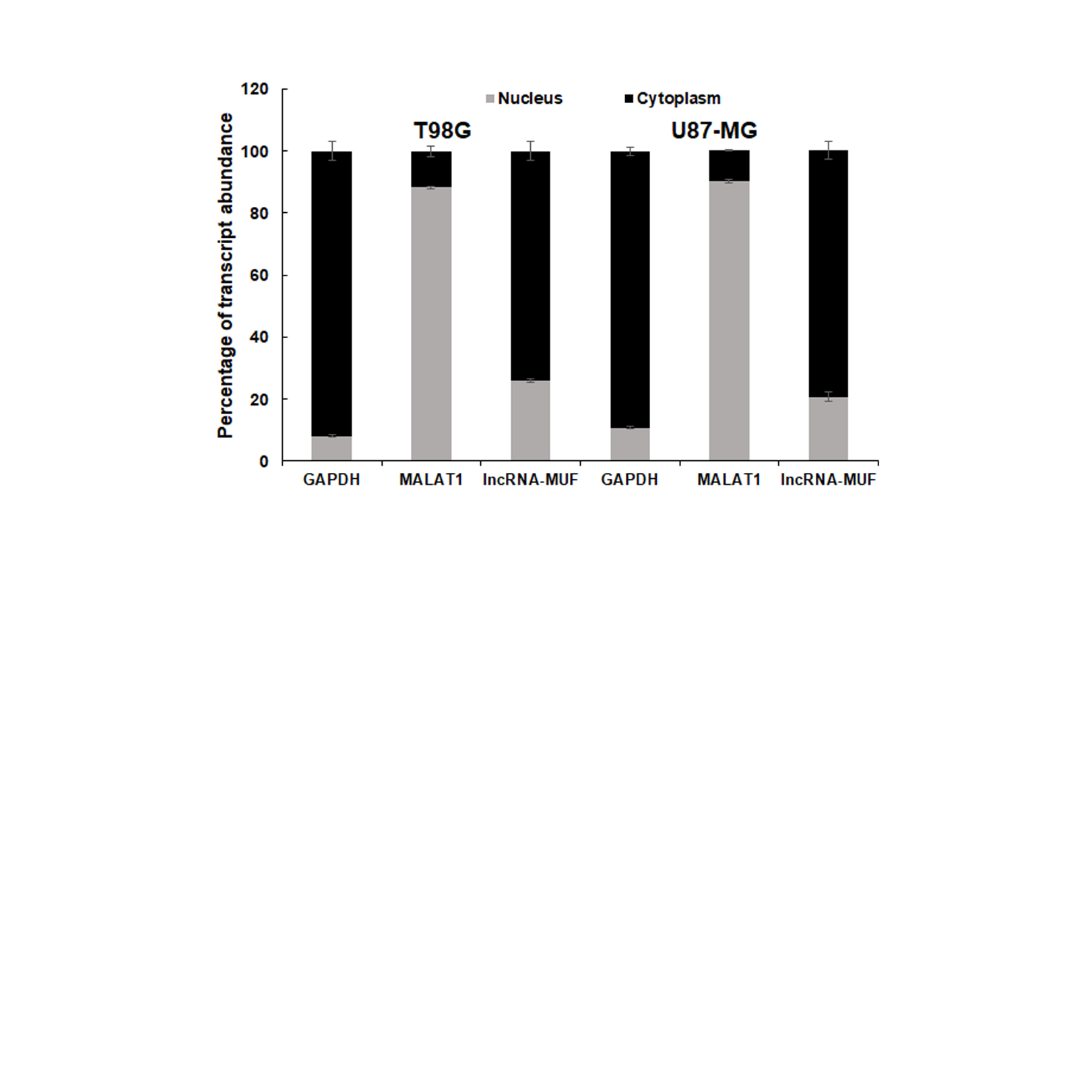
**

LncRNA-MUF is localized primarily in the cytoplasm. GAPDH served as control for cytoplasmic fraction; MALAT1 was used as control for nuclear fraction.

Data are shown as mean ± SD of three independent experiments

**Figure S2C. Luciferase reporter assay for lncRNA-MUF promoter with TGF-β treatment.**


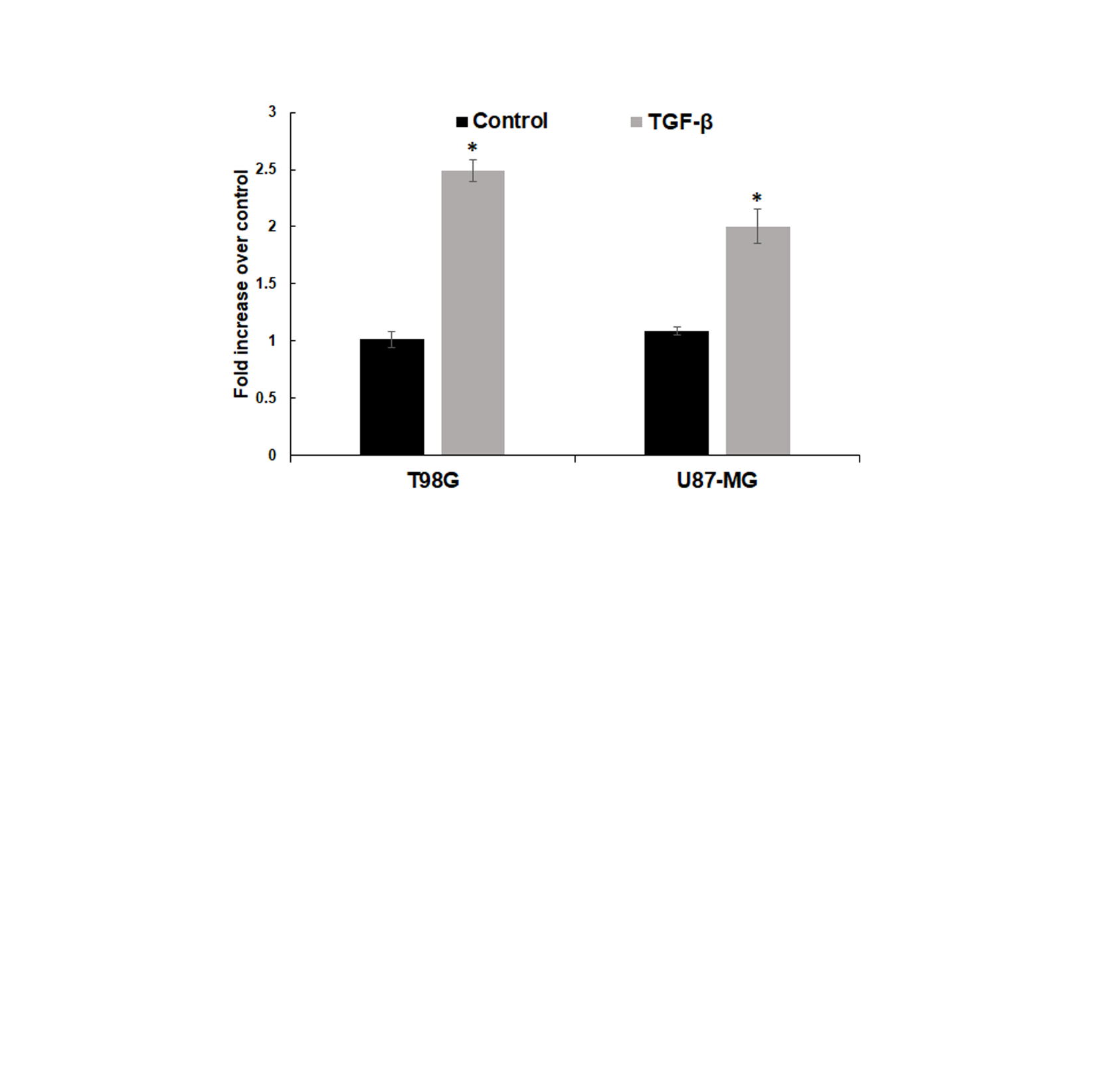


Following transfection of T98G and U87-MG glioma cells with reporter constructs containing -734 bps promoter region of lncRNA-MUF, cells were treated with TGF-β for 24h and the promoter activity was determined.

Data are shown as mean ± SD of three independent experiments

**Figure S3. siRNA mediated knock-down of lncRNA MUF in T98G and U87-MG cells. LncRNA MUF expression was efficiently knocked-down by siRNAs (~ 85% by si-MUF1 and ~ 67 % by si-MUF1) in U87-MG and T98G cells as detected by qRT-PCR assays.**

**
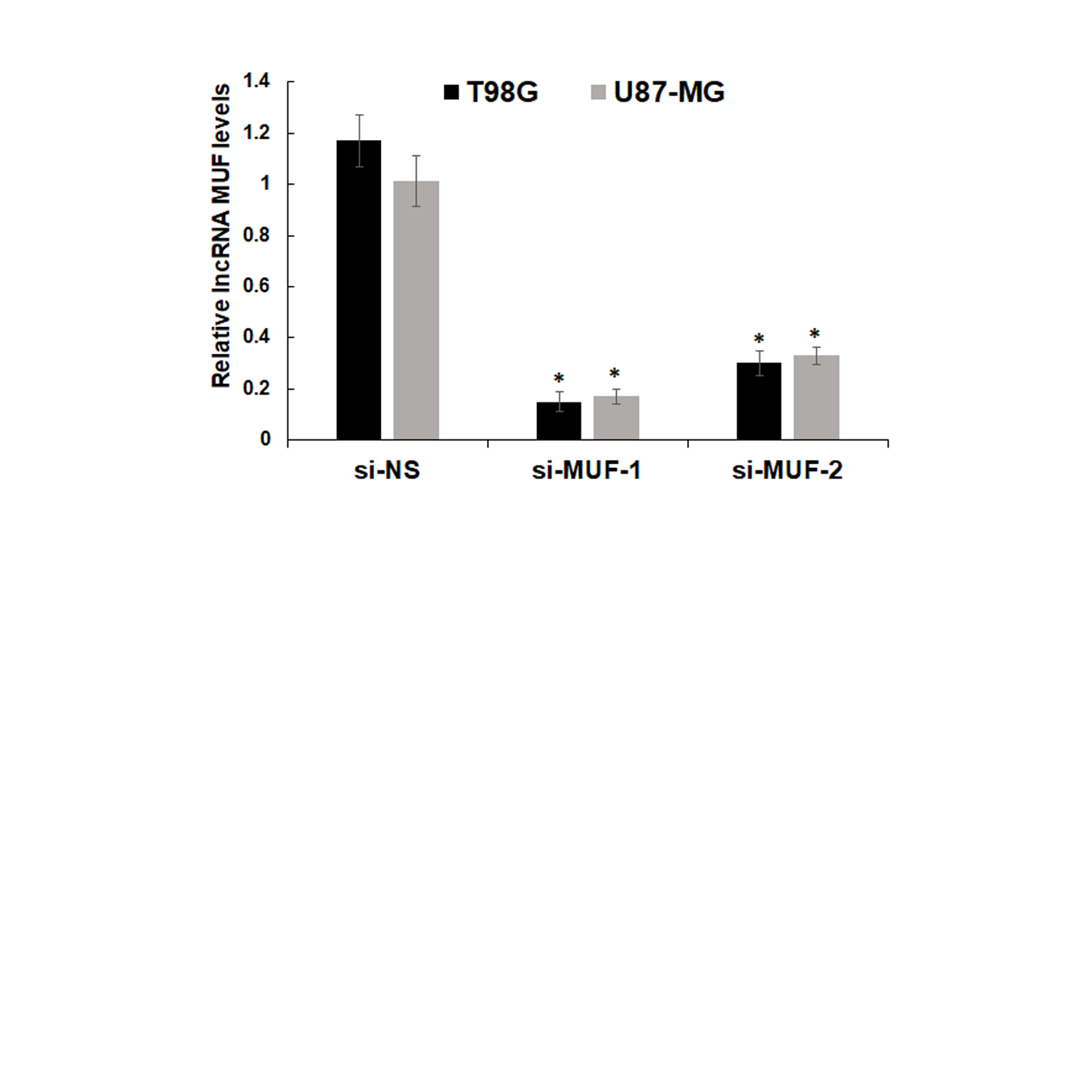
**

siRNA mediated knock-down of lncRNA-MUF in T98G and U87-MG cells. LncRNA-MUF expression was efficiently knocked-down by siRNAs (~ 85% by si-MUF1 and ~ 67 % by si-MUF1) in U87-MG and T98G cells as detected by qRT-PCR assays. Values represent mean ± SEM from three independent experiments. *Significant change compared to cells transfected with control siRNA (si-NS) (p < 0.05).

**Figure S4. Effect of lncRNA-MUF knock-down in glioma cells.**

**
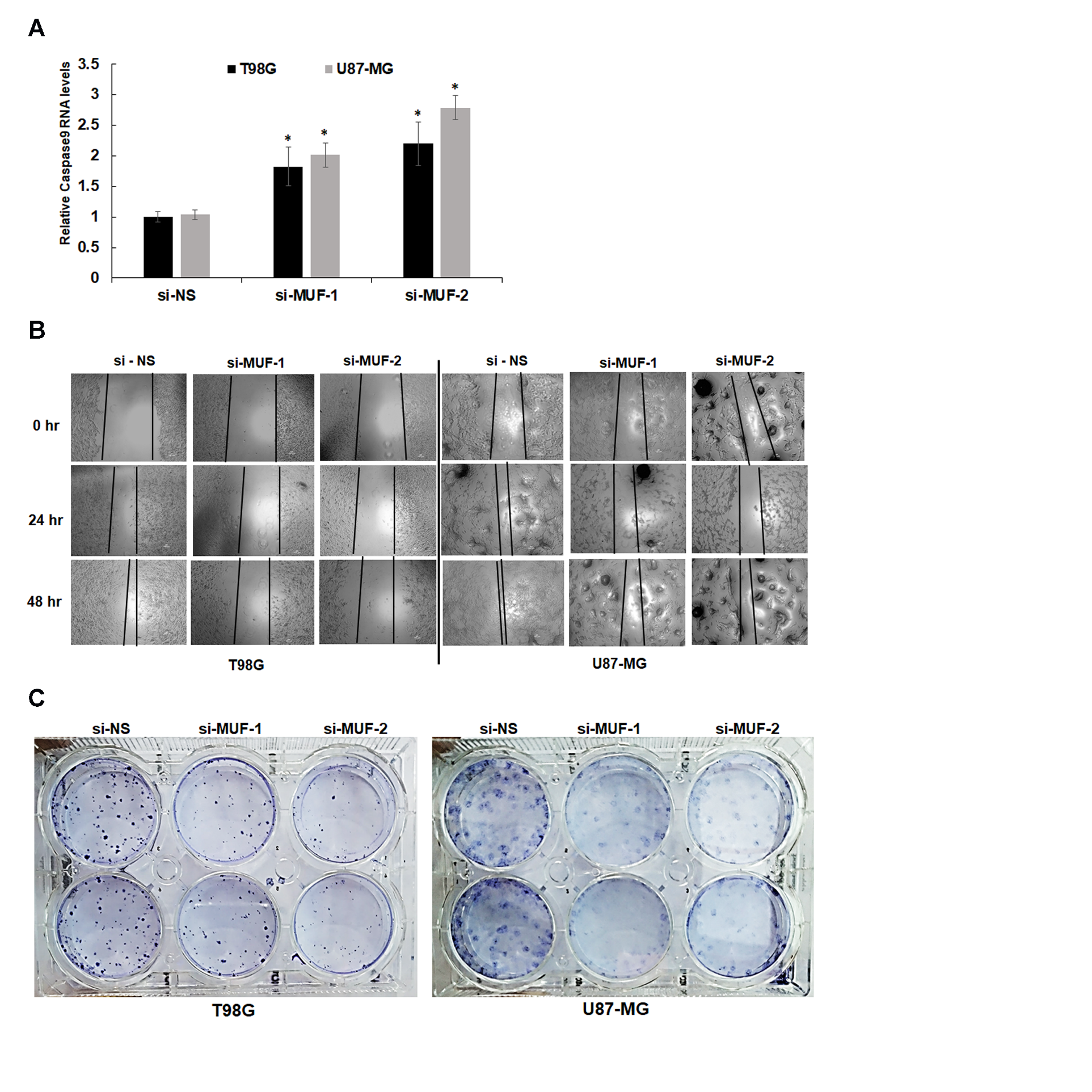
**

1. Knock-down of lncRNA-MUF shows increased caspase 9 mRNA levels in T98G and U87-MG glioma cells measured by qRT-PCR assay.
2. Representative images of reduced migration ability of T98G and U87-MG GBM cells with lncRNA-MUF knock-down.
3. Representative images of reduced colony formation ability of T98G and U87-MG GBM cells with lncRNA-MUF knock-down.

*Significant change compared to cells transfected with control siRNA (si-NS) (p < 0.05).

**Figure S5. Effect of lncRNA-MUF knock-down on cis and trans gene expression.**

**
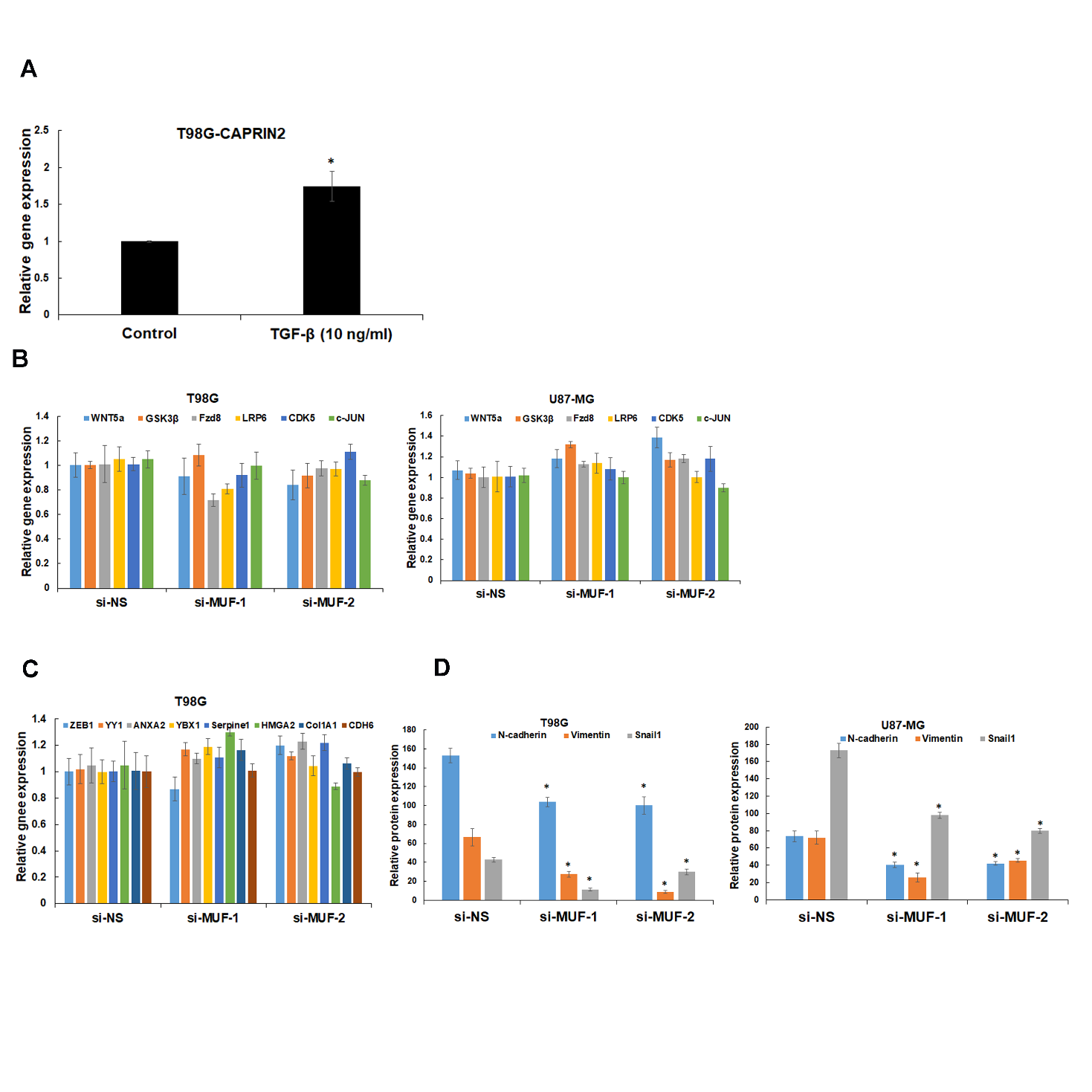
**

1. Relative mRNA levels of Caprin2 gene with TGF-β1 treatment in T98G cells identified by qRT-PCR assay.
2. Gene expression of Wnt/ β-catenin target genes upon lncRNA-MUF knock-down measured by qRT-PCR assays.
3. Gene expression of TGF-β target genes upon lncRNA-MUF knock-down measured by qRT-PCR assays.
4. Quantification of EMT markers protein levels from western blotting upon lncRNA-MUF knock-down using ImageJ software.

**Figure S6. Quantification of psmad2/3 proteins levels from western blotting upon lncRNA-MUF knock-down using ImageJ software.**

**
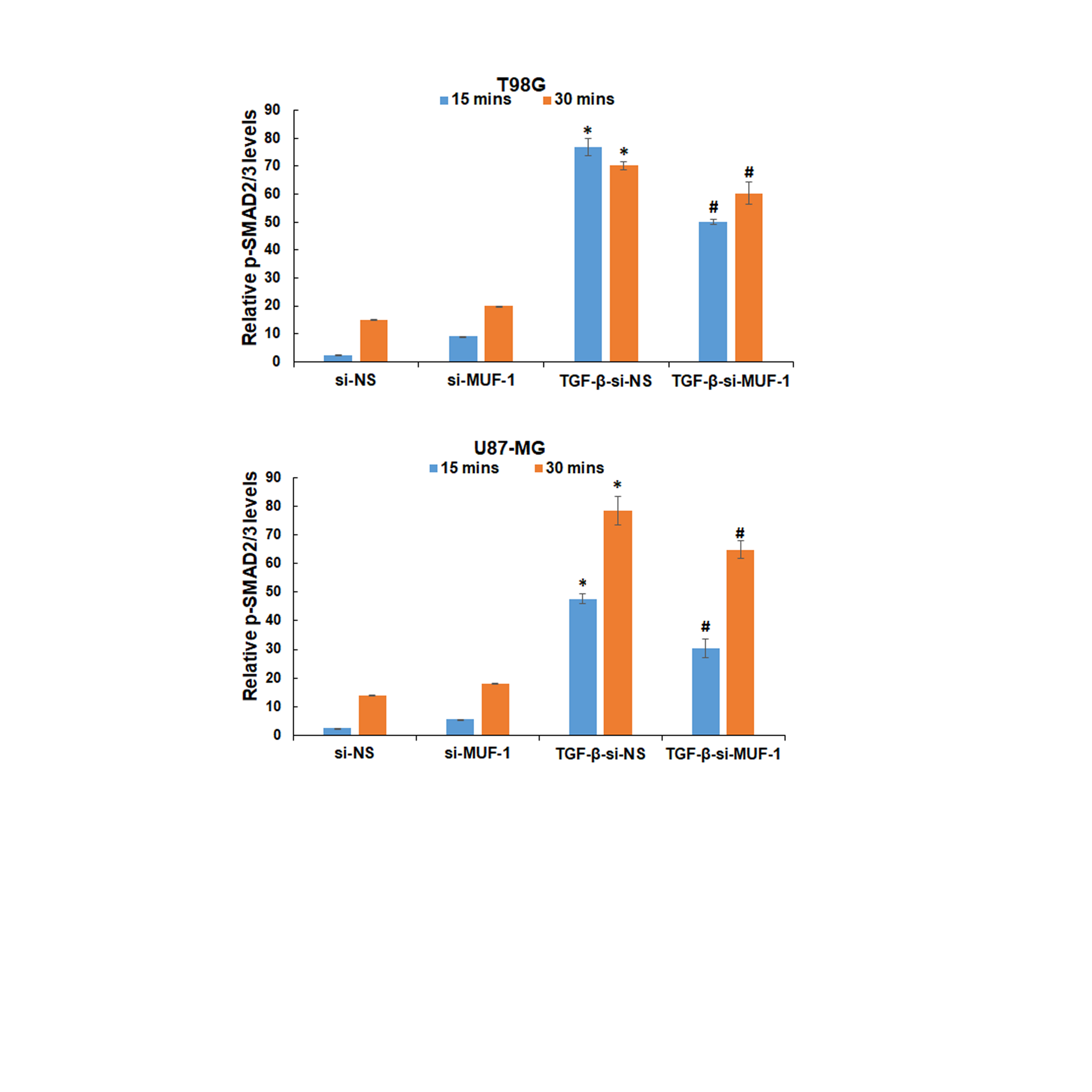
**

psmad2/3 proteins levels analysed by western blotting upon lncRNA-MUF knock-down and TGF-β1 treatment in T98G and U87-MG GBM cells

**Figure S7. LncRNA-MUF sponges miR-34a and promotes Snail1 expression and invasion in glioma.**

**
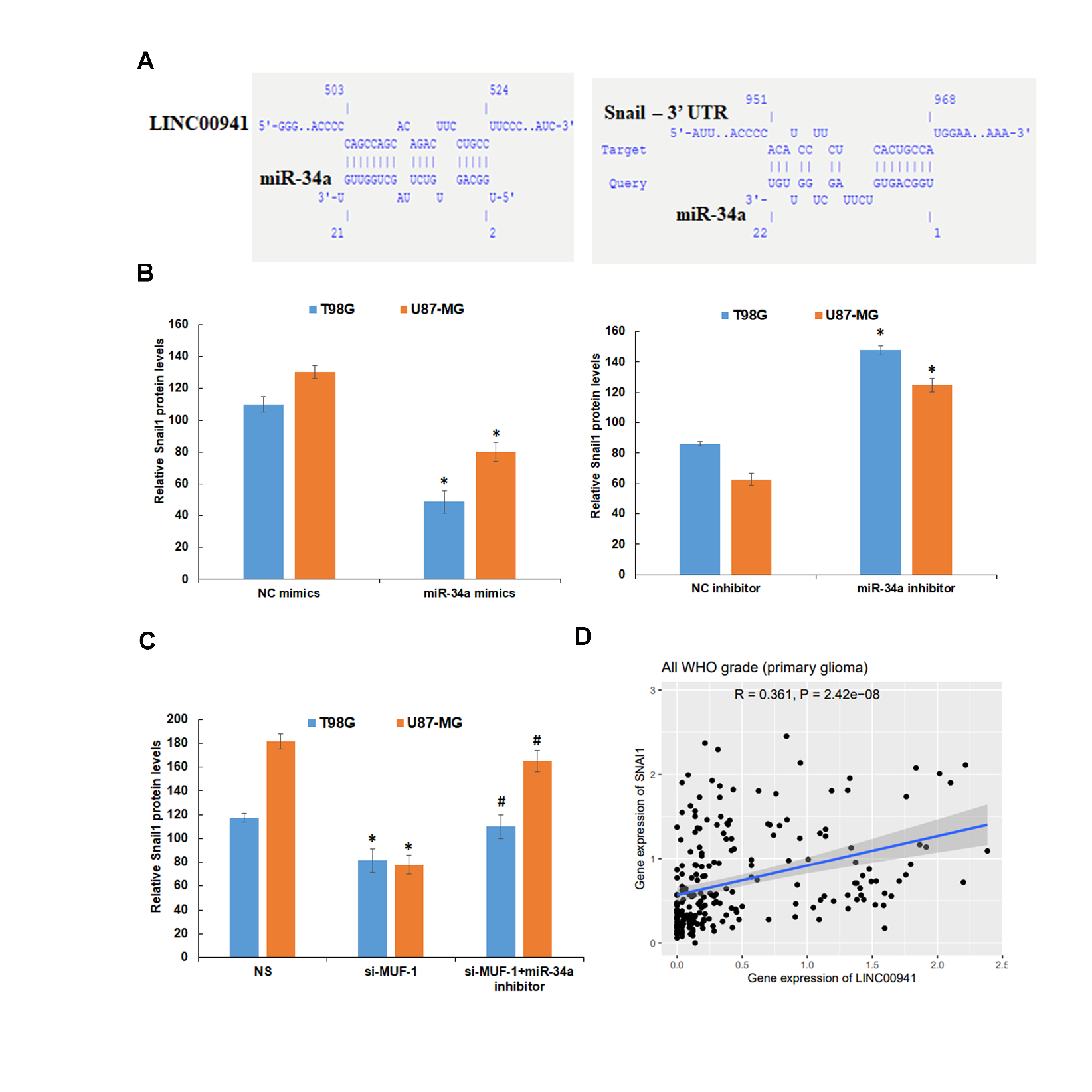
**

1. The predicted miR-34a binding sites on lncRNA-MUF and predicted miR-34a binding sites on Snail1 3’ UTR.
2. Quantification of Snail1 levels from western blotting upon miR-34a mimics and inhibitor transfection using ImageJ software.
3. Quantification of Snail1 levels from western blotting upon si-MUF-1 and miR-34a inhibitor transfection using ImageJ software.
4. Positive correlation between lncRNA-MUF and Snail1 expression in primary GBM samples identified from CGGA dataset (p=2.42^e-08^).

**Supplementary table I : List of primers used for qRT‐PCR**

| LncRNA-MUF-FP1 | CCTCCAACCCCCTTTTCTCC |
| --- | --- |
| LncRNA-MUF-RP1 | GAAGGCAGGAAGTCTGTGCT |
| LncRNA-MUF-FP2 | ACCACTACACTCAGCCAAATAC |
| LncRNA-MUF-RP2 | GGCTATCAACTGTCTCCTTTAGAC |
| ENST00000409910-FP | CACTTGCTTCCTTTGCCACC |
| ENST00000409910-RP | TGCTTTGCCAACCAAAACCA |
| LOC79160-FP | GGAGACCTGTGGTTCTCAGC |
| LOC79160-RP | GAGAGAAACGGCTCCAGACC |
| LINC00312-FP | CTGTGAGAGCAACAGGAGCA |
| LINC00312-RP | TCCTGTGTGAGCTTCCGTTC |
| LOC101928710-FP | ATCCAACACAGTCAGCCAGG |
| LOC101928710-RP | AAGACAGCATAGCCCACACC |
| lnc-EGR2-1-FP | GAGTTGGCGGATGAACACGA |
| lnc-EGR2-1-RP | ATGGCAAGAGCAACTCAGGC |
| CTB-178M22.2-FP | AGAGCAACTGGAGCAAGTCC |
| CTB-178M22.2-RP | GCTGGAAGAGGATAGGCCAC |
| KCNMA1-AS1-FP | TCTTCCCAACCTGCCAAGAC |
| KCNMA1-AS1-RP | GTTCCTCCCAATGTCCCCAG |
| Vimetin-FP | CAGCTAACCAACGACAAA |
| Vimentin-RP | CGTGGAGTTTCTTCAAAAAG |
| SNAI1-FP | CAATCGGAAGCCTAACTA |
| SNAI1-RP | CAGATGAGCATTGGCAGCG |
| CTGF-FP | GTTTGGCCCAGACCCAACTA |
| CTGF-RP | GGCTCTGCTTCTCTAGCCTG |
| cMYC-FP | TCAAGAGGCGAACACACAAC |
| cMYC-RP | GGCCTTTTCATTGTTTTCCA |
| Caspase9-FP | CGAACTAACAGGCAAGCAG |
| Caspase9-RP | ACCTCACCAAATCCTCCAGAAC |
| CAPRIN2-FP | TGCCTGGGGAGTCACCTT |
| CAPRIN2-RP | TGACTGTGGTTCACTTGGGG |
| ANXA2-FP | CTTTCCTGGAGAGGGAGAAATG |
| ANXA2-RP | ATCGCTTTGGCTACTCGTAAA |
| Zeb1-FP | ACTCTGATTCTACACCGC |
| Zeb1-RP | TGTCACATTGATAGGGCTT |
| YY1-FP | ACGGCTTCGAGGATCAGATTC |
| YY1-RP | TGACCAGCGTTTGTTCAATGT |
| YBX1-FP | GGGTGCAGGAGAACAAGGTA |
| YBX1-RP | TCTTCATTGCCGTCCTCTCT |
| Serpine1-FP | GGCTGACTTCACGAGTCTTTCA |
| Serpine1-RP | ATGCGGGCTGAGACTATGACA |
| HMGA2-RP | AGCGCCTCAGAAGAGAGGAC |
| HMGA2-RP | GAGCTGCTTTAGAGGGACTCTTGT |
| Col1A1-FP | CCCCTGGTGCTACTGGTTTCCC |
| Col1A1-RP | GACCTTTGCCGCCTTCTTTGC |
| TBP-FP | GAGCTGTGATGTGAAGTTTCC |
| TBP-RP | TCTGGGTTTGATCATTCTGTAG |
| HPRT-FP | TGAGGATTTGGAAAGGGTGT |
| HPRT-RP | GAGCACACAGAGGGCTACAA |
| JUNB-FP | ACTCATACACAGCTACGGGATACG |
| JUNB-RP | GGCTCGGTTTCAGGAGTTTG |
| MMP10-FP | GTCTCTCTACGGACCTCCCC |
| MMP10-RP | GGGTTCCAGTGGGATCTTCG |
| MMP13-FP | TGGCTGCCTTCCTCTTCTTG |
| MMP13-RP | GCTCTGCAAACTGGAGGTCT |
| LAMP3-FP | GGAAATGTGGATGAGTGCTCG |
| LAMP3-RP | CCCCCGGGCAACAATTAGAT |
| TNFAIP6-FP | GCTACAACCCACACGCAAAG |
| TNFAIP6-RP | CCAAGCAACCTGGGTCATCT |
| EGR2-FP | CAGGAGAGAGTCAGTGGCAAA |
| EGR2-RP | CTCGCTACCTGGAGTGTCAG |
| Lefty-FP | AGAGCTGGCGATGACTGAAC |
| Lefty-RP | AAACTGAGCAAGGGCTCTCC |
| KCNB1-FP | CCCGCTACCACCAGAAGAAA |
| KCNB1-RP | TGGCAAGGATCTTGGCAGC |
| KIT-FP | TACTTGGAGCCTGCACCATT |
| KIT-RP | TATCGCTGCAGGAAGACTCC |
| TGFbR3-FP | CAGGAGGTGAGAGTCCCCAG |
| TGFbR3-RP | GCAATTTTCAAACTGCCTCGG |
| WNT5a-FP | AGGGCTCCTACGAGAGTGCT |
| WNT5a-RP | GACACCCCATGGCACTTG |
| GSK3b-FP | TCGAGAGCTCCAGATCATGAGAA |
| GSK3b-RP | CGGAACATAGTCCAGCACCAGA |
| Fzd8-FP | GCTCTACAACCGCGTCAAGA |
| Fzd8-RP | GCTGAAAAAGGGGTTGTGGC |
| LRP6-FP | TTGTTGCTTTATGCAAACAGACG |
| LRP6-RP | CGTTTAATGGCTTCTTCGCTGAC |
| CDK5-FP | GGGAAGGCACCTACGGAACTG |
| CDK5-RP | GGCGGAACTCGGCACACC |
| c-JUN-FP | TCCAAGTGCCGAAAAAGGAAG |
| c-JUN-RP | CGAGTTCTGAGCTTTCAAGGT |
| IPO8-FP | CATGATGCCTCTCCTGCATAA |
| IPO8-RP | CTTCTCCTGCATCTCCACATAG |
| LOC645485-FP | AAGATGCTCCTGCCACCTTC |
| LOC645485-RP | GTCAAAACGCCAGAGGGAGA |
| LOC107984476-FP | CAATGTCTCGCATGAAGCGG |
| LOC107984476-RP | AGCTCTTTCCAGATGTGCCC |
| miR-34a-5p-FP | TGGCAGTGTCTTAGCTGGTTGT |
| U6-FP | CTCGCTTCGGCAGCACA |
| U6-RP | AACGCTTCACGAATTTGCGT |

**Supplementary table II : List of differentially expressed lncRNAs upon TGF-β1 (10 ng/ml) treatment in T98G GBM cells identified by microarray screen**

| **S.No** | **ID** | **Fold change** | **p value** | **NCBI ENSEMBL Annotations** |
| --- | --- | --- | --- | --- |
| 1 | ENST00000409910 | **6.732** | **0.025** | ens\|ENST00000409910\|linc\|lnc-AC112721.1.1-1:1\|linc\|TCONS_00003518\|gb\|DA144367 |
| 2 | lnc-CLEC18B-3 | **4.287** | **0.002** | linc\|lnc-CLEC18B-3:6\|linc\|TCONS_00025051 |
| 3 | ENST00000435044 | **4.127** | **0.000** | ens\|ENST00000435044\|linc\|lnc-RP11-90M5.1.1-3:1\|linc\|TCONS_l2_00007378\|linc\|TCONS_l2_00006770 |
| 4 | LOC79160 | **3.989** | **0.000** | ref\|NR_125359\|ens\|ENST00000598340\|gb\|BC002831\|tc\|THC2483328 |
| 5 | LINC01049 | **3.609** | **0.005** | ref\|NR_120414\|linc\|lnc-GPC5-3:2\|linc\|TCONS_00021501\|gb\|BC038529 |
| 6 | LINC00312 | **3.551** | **0.000** | ref\|NR_024065\|ens\|ENST00000442796\|linc\|lnc-LMCD1-1:1\|linc\|lnc-LMCD1-1:2 |
| 7 | ENST00000563424 | **3.379** | **0.000** | ens\|ENST00000563424\|linc\|lnc-NKD1-1:1\|tc\|THC2652817 |
| 8 | lnc-MSRB3-2 | **3.336** | **0.001** | linc\|lnc-MSRB3-2:2\|linc\|TCONS_00020456 |
| 9 | FAM222A-AS1 | **3.292** | **0.023** | ref\|NR_026661\|ref\|NR_026662\|linc\|lnc-TRPV4-1:2\|linc\|lnc-TRPV4-1:1 |
| 10 | LINC01405 | **3.264** | **0.001** | ens\|ENST00000553177\|linc\|lnc-CCDC63-2:1\|linc\|lnc-CCDC63-2:8\|linc\|TCONS_00020561 |
| 11 | lnc-CNIH3-1 | **3.126** | **0.020** | linc\|lnc-CNIH3-1:2\|linc\|TCONS_00000410 |
| 12 | lnc-FSIP1-1 | **2.949** | **0.000** | linc\|lnc-FSIP1-1:1 |
| 13 | lnc-AIRE-1 | **2.930** | **0.001** | linc\|lnc-AIRE-1:1\|tc\|THC2688962 |
| 14 | lnc-CHAF1B-2 | **2.833** | **0.004** | linc\|lnc-CHAF1B-2:1 |
| 15 | ENST00000511422 | **2.800** | **0.019** | ens\|ENST00000511422\|linc\|lnc-PRR16-1:3\|linc\|TCONS_00009497 |
| 16 | lnc-CLCN3-3 | **2.789** | **0.034** | linc\|lnc-CLCN3-3:1\|gb\|AW966219\|tc\|THC2560866 |
| 17 | ENST00000539116 | **2.738** | **0.010** | ens\|ENST00000539116\|ens\|ENST00000541391\|linc\|lnc-MSRB3-2:3\|linc\|lnc-MSRB3-2:1 |
| 18 | LOC101927253 | **2.660** | **0.032** | ref\|XR_243718\|ref\|XR_253693\|linc\|lnc-ANKRD40-2:1\|gb\|AK000454 |
| 19 | lnc-COX10-1 | **2.624** | **0.000** | linc\|lnc-COX10-1:2\|linc\|lnc-COX10-1:1\|linc\|TCONS_00025844\|linc\|TCONS_00025311 |
| 20 | ENST00000564772 | **2.596** | **0.003** | ens\|ENST00000564772\|linc\|lnc-TPST2-1:1\|gb\|AK093872\|tc\|THC2557975 |
| 21 | LOC101928710 | **2.593** | **0.000** | ref\|NR_110848\|ref\|NR_110849\|ens\|ENST00000591384\|ens\|ENST00000590023 |
| 22 | lnc-EGR2-1 | **2.565** | **0.001** | linc\|lnc-EGR2-1:1 |
| 23 | lnc-RP11-597K23.2.1-2 | **2.448** | **0.024** | linc\|lnc-RP11-597K23.2.1-2:1\|linc\|TCONS_00023486\|tc\|THC2616036 |
| 24 | XLOC_l2_010963 | **2.440** | **0.004** | linc\|TCONS_l2_00020917\|linc\|TCONS_l2_00020919 |
| 25 | TMEM92-AS1 | **2.403** | **0.001** | ref\|NR_125805\|linc\|lnc-COL1A1-4:3\|linc\|lnc-COL1A1-4:2\|linc\|TCONS_00025694 |
| 26 | LOC102725378 | **2.394** | **0.001** | ref\|XR_425288\|ref\|XR_433664\|linc\|lnc-C21orf33-3:1\|linc\|TCONS_00029050 |
| 27 | A_33_P3247072 | **2.284** | **0.000** |  |
| 28 | LOC102723834 | **2.105** | **0.000** | ref\|XR_425103\|ref\|XR_426933\|ref\|XR_431366\|linc\|lnc-SRP9-1:2 |
| 29 | lnc-ZNF643-1 | **2.075** | **0.024** | linc\|lnc-ZNF643-1:1\|gb\|AK094134 |
| 30 | lnc-NACA2-2 | **2.008** | **0.047** | linc\|lnc-NACA2-2:1\|linc\|TCONS_00025725 |
| 31 | lnc-RCL1-1 | **2.007** | **0.015** | linc\|lnc-RCL1-1:1\|gb\|AK021739 |
| 32 | ENST00000418271 | **1.955** | **0.042** | ens\|ENST00000418271\|ens\|ENST00000444114\|linc\|lnc-MN1-6:2\|linc\|lnc-MN1-6:1 |
| 33 | LINC01137 | **1.942** | **0.000** | ref\|NR_038842\|linc\|lnc-MEAF6-1:2\|linc\|lnc-MEAF6-1:3\|linc\|lnc-MEAF6-1:1 |
| 34 | LOC100130417 | **1.922** | **0.002** | ref\|NR_026874\|ref\|NR_122045\|ens\|ENST00000432961\|ens\|ENST00000417705 |
| 35 | ENST00000444114 | **1.914** | **0.002** | ens\|ENST00000444114\|linc\|lnc-MN1-6:1\|linc\|TCONS_l2_00018380 |
| 36 | ENST00000576365 | **1.882** | **0.015** | ens\|ENST00000576365\|linc\|lnc-CAPNS2-1:1\|ref\|XM_005256006\|gb\|AK126523 |
| 37 | LINC01546 | **1.876** | **0.005** | ref\|NR_038428\|ens\|ENST00000457435\|gb\|BX101503\|tc\|THC2639452 |
| 38 | lnc-ICOSLG-2 | **1.853** | **0.001** | linc\|lnc-ICOSLG-2:1\|linc\|TCONS_00029175 |
| 39 | lnc-CCNB2-1 | **1.833** | **0.011** | linc\|lnc-CCNB2-1:1 |
| 40 | lnc-KCNN4-1 | **1.810** | **0.002** | linc\|lnc-KCNN4-1:1\|ref\|XM_006723156\|ref\|XM_006723157\|linc\|TCONS_00027758 |
| 41 | lnc-SRP9-1 | **1.759** | **0.001** | linc\|lnc-SRP9-1:3\|linc\|lnc-SRP9-1:2\|linc\|lnc-SRP9-1:4\|linc\|TCONS_00001307 |
| 42 | lnc-FAM43A-2 | **1.746** | **0.027** | linc\|lnc-FAM43A-2:1\|tc\|THC2641907 |
| 43 | LINC00941 | **1.735** | **0.004** | ref\|NR_040245\|ens\|ENST00000547804\|linc\|lnc-TSPAN11-2:4\|linc\|lnc-TSPAN11-2:3 |
| 44 | FAM226A | **1.730** | **0.001** | ref\|NR_026595\|gb\|DQ891637\|gb\|DQ894827\|tc\|THC2476986 |
| 45 | FOXP4-AS1 | **1.695** | **0.000** | ref\|NR_126415\|ref\|NR_126417\|ref\|NR_126416\|linc\|lnc-TFEB-1:2 |
| 46 | ENST00000504578 | **1.693** | **0.001** | ens\|ENST00000504578\|linc\|lnc-CTD-2215E18.1.1-1:1\|tc\|THC2704117 |
| 47 | DKFZp434J0226 | **1.684** | **0.002** | ens\|ENST00000593980\|linc\|lnc-IGFL1-1:2\|linc\|lnc-IGFL1-1:3\|linc\|TCONS_00026793 |
| 48 | ENST00000507391 | **1.631** | **0.049** | ens\|ENST00000507391\|linc\|lnc-TCERG1-1:1\|linc\|TCONS_00009536 |
| 49 | ENST00000541888 | **1.607** | **0.005** | ens\|ENST00000541888\|gb\|XR_111249\|tc\|THC2689905 |
| 50 | LOC100507002 | **1.527** | **0.000** | ref\|NR_110801\|ens\|ENST00000582940\|ref\|XR_243742\|ref\|XR_248417 |
| 51 | LOC102724348 | **0.636** | **0.001** | ref\|PREDICTED: Homo sapiens uncharacterized LOC102724348 (LOC102724348), ncRNA [XR_429392] |
| 52 | DKFZP586I1420 | **0.633** | **0.004** | ref\|Homo sapiens uncharacterized protein DKFZp586I1420 (DKFZP586I1420), non-coding RNA [NR_002186] |
| 53 | ENST00000511928 | **0.629** | **0.004** | gb\|Homo sapiens mRNA; cDNA DKFZp686E16130 (from clone DKFZp686E16130). [BX648772] |
| 54 | lnc-SRBD1-1 | **0.616** | **0.001** | linc\|LNCipedia lincRNA (lnc-SRBD1-1), lincRNA [lnc-SRBD1-1:1] |
| 55 | PXN-AS1 | **0.603** | **0.004** | ref\|Homo sapiens PXN antisense RNA 1 (PXN-AS1), long non-coding RNA [NR_038924] |
| 56 | THC2651904 | **0.599** | **0.000** | tc\|Q4SEQ2_TETNG (Q4SEQ2) Chromosome 3 SCAF14614, whole genome shotgun sequence. (Fragment), partial (28%) [THC2651904] |
| 57 | lnc-RAP1GDS1-3 | **0.598** | **0.003** | linc\|LNCipedia lincRNA (lnc-RAP1GDS1-3), lincRNA [lnc-RAP1GDS1-3:4] |
| 58 | PRKAG2-AS1 | **0.593** | **0.001** | ref\|Homo sapiens PRKAG2 antisense RNA 1 (PRKAG2-AS1), long non-coding RNA [NR_038926] |
| 59 | ENST00000623174 | **0.588** | **0.011** | ref\|PREDICTED: Homo sapiens uncharacterized LOC645967 (LOC645967), misc_RNA [XR_245616] |
| 60 | LOC729950 | **0.579** | **0.006** | ref\|Homo sapiens uncharacterized LOC729950 (LOC729950), long non-coding RNA [NR_040033] |
| 61 | lnc-SNRNP200-1 | **0.574** | **0.005** | linc\|LNCipedia lincRNA (lnc-SNRNP200-1), lincRNA [lnc-SNRNP200-1:1] |
| 62 | LOC101929027 | **0.559** | **0.000** | ref\|PREDICTED: Homo sapiens uncharacterized LOC101929027 (LOC101929027), ncRNA [XR_242942] |
| 63 | lnc-OSBPL10-1 | **0.521** | **0.003** | gb\|Homo sapiens cDNA clone IMAGE:5259414. [BC035064] |
| 64 | GHRLOS | **0.520** | **0.011** | ref\|Homo sapiens ghrelin opposite strand/antisense RNA (GHRLOS), transcript variant 5, long non-coding RNA [NR_073566] |
| 65 | LOC101929626 | **0.514** | **0.036** | ref\|Homo sapiens uncharacterized LOC101929626 (LOC101929626), long non-coding RNA [NR_125987] |
| 66 | ENST00000520544 | **0.508** | **0.047** | Unknown |
| 67 | lnc-AHR-2 | **0.498** | **0.006** | linc\|LNCipedia lincRNA (lnc-AHR-2), lincRNA [lnc-AHR-2:1] |
| 68 | ENST00000445534 | **0.497** | **0.004** | Unknown |
| 69 | LOC101927415 | **0.487** | **0.000** | ref\|Homo sapiens uncharacterized LOC101927415 (LOC101927415), long non-coding RNA [NR_110049] |
| 70 | lnc-KLRG2-1 | **0.477** | **0.000** | gb\|Homo sapiens mRNA; cDNA DKFZp451B0818 (from clone DKFZp451B0818). [AL832624] |
| 71 | lnc-THNSL1-2 | **0.476** | **0.006** | gb\|Homo sapiens cDNA FLJ11983 fis, clone HEMBB1001337. [AK022045] |
| 72 | ENST00000527434 | **0.454** | **0.004** | ens\|WASH and IL9R antisense RNA 2 [Source:HGNC Symbol;Acc:HGNC:38609] [ENST00000527434] |
| 73 | A_33_P3227457 | **0.450** | **0.002** | A_33_P3227457 |
| 74 | AK002210 | **0.450** | **0.024** | gb\|Homo sapiens cDNA FLJ11348 fis, clone PLACE4000638. [AK002210] |
| 75 | ZNF295-AS1 | **0.427** | **0.000** | ref\|Homo sapiens ZNF295 antisense RNA 1 (ZNF295-AS1), transcript variant 1, long non-coding RNA [NR_119384] |
| 76 | THC2548955 | **0.424** | **0.009** | THC2548955 |
| 77 | A_23_P90470 | **0.424** | **0.033** | A_23_P90470 |
| 78 | lnc-CHSY1-5 | **0.393** | **0.012** | linc\|LNCipedia lincRNA (lnc-CHSY1-5), lincRNA [lnc-CHSY1-5:4] |
| 79 | LINC01564 | **0.338** | **0.058** | ref\|Homo sapiens long intergenic non-protein coding RNA 1564 (LINC01564), long non-coding RNA [NR_125841] |
| 80 | lnc-TIAL1-1 | **0.331** | **0.013** | gb\|602076586F1 NIH_MGC_62 Homo sapiens cDNA clone IMAGE:4243748 5', mRNA sequence [BF572570] |
| 81 | lnc-SOX6-1 | **0.314** | **0.048** | linc\|LNCipedia lincRNA (lnc-SOX6-1), lincRNA [lnc-SOX6-1:1] |
| 82 | lnc-TCL1B-2 | **0.302** | **0.025** | gb\|human full-length cDNA clone CS0DG006YJ19 of B cells (Ramos cell line) of Homo sapiens (human). [BX247990] |
| 83 | lnc-RPP30-2 | **0.299** | **0.044** | linc\|LNCipedia lincRNA (lnc-RPP30-2), lincRNA [lnc-RPP30-2:1] |
| 84 | lnc-DYDC1-4 | **0.287** | **0.047** | tc\|Q4NC16_9MICC (Q4NC16) Binding-protein-dependent transport systems inner membrane component, partial (5%) [THC2722395] |
| 85 | LOC101928152 | **0.253** | **0.000** | ref\|PREDICTED: Homo sapiens uncharacterized LOC101928152 (LOC101928152), ncRNA [XR_245037] |
| 86 | lnc-SUPT3H-1 | **0.245** | **0.024** | linc\|LNCipedia lincRNA (lnc-SUPT3H-1), lincRNA [lnc-SUPT3H-1:3] |
| 87 | lnc-HMCN1-2_TTDlnc5 | **0.242** | **0.045** | linc\|LNCipedia lincRNA (lnc-HMCN1-2), lincRNA [lnc-HMCN1-2:1] |
| 88 | PARD3-AS1 | **0.238** | **0.000** | ref\|Homo sapiens PARD3 antisense RNA 1 (PARD3-AS1), long non-coding RNA [NR_108043] |
| 89 | lnc-TMEM64-2 | **0.212** | **0.016** | gb\|by03b05.y1 Human Lens cDNA (Un-normalized, unamplified): BY Homo sapiens cDNA clone by03b05 5', mRNA sequence [BF726218] |
| 90 | KCNMA1-AS1 | **0.182** | **0.005** | ref\|PREDICTED: Homo sapiens uncharacterized LOC101929328 (LOC101929328), ncRNA [XR_246137] |
| 91 | CTB-178M22.2 | **0.157** | **0.001** | ref\|Homo sapiens uncharacterized LOC101927862 (CTB-178M22.2), long non-coding RNA [NR_109894] |

**List of differentially expressed mRNAs upon TGF-β1 (10 ng/ml) treatment in T98G GBM cells identified by microarray screen**

| **S.No** | **Gene** | **Fold change** | **p value** | **NCBI/ ENSEMBL ID** | **Annotations** |
| --- | --- | --- | --- | --- | --- |
| 1 | GPA33 | **19.790** | **0.000** | NM_005814 | ref\|Homo sapiens glycoprotein A33 (transmembrane) (GPA33), mRNA [NM_005814] |
| 2 | SERPINE1 | **18.523** | **0.000** | NM_000602 | ref\|Homo sapiens serpin peptidase inhibitor, clade E (nexin, plasminogen activator inhibitor type 1), member 1 (SERPINE1), mRNA [NM_000602] |
| 3 | FOXS1 | **16.556** | **0.000** | NM_004118 | ref\|Homo sapiens forkhead box S1 (FOXS1), mRNA [NM_004118] |
| 4 | LEFTY1 | **15.429** | **0.000** | NM_020997 | ref\|Homo sapiens left-right determination factor 1 (LEFTY1), mRNA [NM_020997] |
| 5 | EGR2 | **13.478** | **0.000** | NM_000399 | ref\|Homo sapiens early growth response 2 (EGR2), transcript variant 1, mRNA [NM_000399] |
| 6 | COL1A1 | **13.325** | **0.000** | NM_000088 | ref\|Homo sapiens collagen, type I, alpha 1 (COL1A1), mRNA [NM_000088] |
| 7 | AMTN | **13.241** | **0.002** | NM_212557 | ref\|Homo sapiens amelotin (AMTN), transcript variant 1, mRNA [NM_212557] |
| 8 | MFAP2 | **13.057** | **0.000** | NM_017459 | ref\|Homo sapiens microfibrillar-associated protein 2 (MFAP2), transcript variant 1, mRNA [NM_017459] |
| 9 | RPS21 | **12.287** | **0.000** | ENST00000492356 | ens\|ribosomal protein S21 [Source:HGNC Symbol;Acc:HGNC:10409] [ENST00000492356] |
| 10 | BTBD16 | **11.422** | **0.001** | NM_144587 | ref\|Homo sapiens BTB (POZ) domain containing 16 (BTBD16), mRNA [NM_144587] |
| 11 | PMEPA1 | **10.922** | **0.000** | NM_020182 | ref\|Homo sapiens prostate transmembrane protein, androgen induced 1 (PMEPA1), transcript variant 1, mRNA [NM_020182] |
| 12 | LAMP3 | **10.229** | **0.000** | NM_014398 | ref\|Homo sapiens lysosomal-associated membrane protein 3 (LAMP3), mRNA [NM_014398] |
| 13 | TSPAN2 | **10.220** | **0.003** | NM_005725 | ref\|Homo sapiens tetraspanin 2 (TSPAN2), mRNA [NM_005725] |
| 14 | APCDD1L | **10.210** | **0.000** | NM_153360 | ref\|Homo sapiens adenomatosis polyposis coli down-regulated 1-like (APCDD1L), mRNA [NM_153360] |
| 15 | IL11 | **9.894** | **0.005** | NM_000641 | ref\|Homo sapiens interleukin 11 (IL11), transcript variant 1, mRNA [NM_000641] |
| 16 | HAVCR2 | **8.430** | **0.005** | NM_032782 | ref\|Homo sapiens hepatitis A virus cellular receptor 2 (HAVCR2), mRNA [NM_032782] |
| 17 | PLEKHG1 | **7.515** | **0.001** | NM_001029884 | ref\|Homo sapiens pleckstrin homology domain containing, family G (with RhoGef domain) member 1 (PLEKHG1), mRNA [NM_001029884] |
| 18 | TNFAIP6 | **7.406** | **0.001** | NM_007115 | ref\|Homo sapiens tumor necrosis factor, alpha-induced protein 6 (TNFAIP6), mRNA [NM_007115] |
| 19 | MYOZ1 | **6.816** | **0.003** | NM_021245 | ref\|Homo sapiens myozenin 1 (MYOZ1), mRNA [NM_021245] |
| 20 | MMP10 | **6.637** | **0.019** | NM_002425 | ref\|Homo sapiens matrix metallopeptidase 10 (stromelysin 2) (MMP10), mRNA [NM_002425] |
| 21 | SFRP4 | **6.551** | **0.010** | NM_003014 | ref\|Homo sapiens secreted frizzled-related protein 4 (SFRP4), mRNA [NM_003014] |
| 22 | PDGFB | **6.408** | **0.002** | NM_002608 | ref\|Homo sapiens platelet-derived growth factor beta polypeptide (PDGFB), transcript variant 1, mRNA [NM_002608] |
| 23 | MMP13 | **6.282** | **0.000** | NM_002427 | ref\|Homo sapiens matrix metallopeptidase 13 (collagenase 3) (MMP13), mRNA [NM_002427] |
| 24 | F2RL1 | **6.254** | **0.000** | NM_005242 | ref\|Homo sapiens coagulation factor II (thrombin) receptor-like 1 (F2RL1), mRNA [NM_005242] |
| 25 | SERPINE2 | **6.136** | **0.000** | NM_006216 | ref\|Homo sapiens serpin peptidase inhibitor, clade E (nexin, plasminogen activator inhibitor type 1), member 2 (SERPINE2), transcript variant 1, mRNA [NM_006216] |
| 26 | ENST00000390547 | **5.931** | **0.010** | ENST00000390547 | ens\|immunoglobulin heavy constant alpha 1 [Source:HGNC Symbol;Acc:HGNC:5478] [ENST00000390547] |
| 27 | RGS16 | **5.742** | **0.000** | NM_002928 | ref\|Homo sapiens regulator of G-protein signaling 16 (RGS16), mRNA [NM_002928] |
| 28 | JUNB | **5.302** | **0.000** | NM_002229 | ref\|Homo sapiens jun B proto-oncogene (JUNB), mRNA [NM_002229] |
| 29 | PROC | **5.132** | **0.000** | NM_000312 | ref\|Homo sapiens protein C (inactivator of coagulation factors Va and VIIIa) (PROC), mRNA [NM_000312] |
| 30 | MOV10L1 | **5.130** | **0.000** | NM_018995 | ref\|Homo sapiens Mov10 RISC complex RNA helicase like 1 (MOV10L1), transcript variant 1, mRNA [NM_018995] |
| 31 | SLC29A1 | **5.035** | **0.000** | NM_001078177 | ref\|Homo sapiens solute carrier family 29 (equilibrative nucleoside transporter), member 1 (SLC29A1), transcript variant 1, mRNA [NM_001078177] |
| 32 | RGS9 | **5.019** | **0.000** | NM_003835 | ref\|Homo sapiens regulator of G-protein signaling 9 (RGS9), transcript variant 1, mRNA [NM_003835] |
| 33 | KCNK12 | **4.741** | **0.003** | NM_022055 | ref\|Homo sapiens potassium channel, two pore domain subfamily K, member 12 (KCNK12), mRNA [NM_022055] |
| 34 | CCL7 | **4.735** | **0.011** | NM_006273 | ref\|Homo sapiens chemokine (C-C motif) ligand 7 (CCL7), mRNA [NM_006273] |
| 35 | AK098835 | **4.603** | **0.041** | AK098835 | gb\|Homo sapiens cDNA FLJ25969 fis, clone CBR02250. [AK098835] |
| 36 | RET | **4.380** | **0.003** | NM_020630 | ref\|Homo sapiens ret proto-oncogene (RET), transcript variant 4, mRNA [NM_020630] |
| 37 | ENST00000508366 | **4.253** | **0.001** | ENST00000508366 | ens\|ATPase, H+ transporting, lysosomal accessory protein 1-like [Source:HGNC Symbol;Acc:HGNC:28091] [ENST00000508366] |
| 38 | ARHGAP22 | **4.246** | **0.000** | NM_021226 | ref\|Homo sapiens Rho GTPase activating protein 22 (ARHGAP22), transcript variant 3, mRNA [NM_021226] |
| 39 | ZNF468 | **4.242** | **0.000** | NM_001277120 | ref\|Homo sapiens zinc finger protein 468 (ZNF468), transcript variant 1, mRNA [NM_001277120] |
| 40 | MIR181A2HG | **4.211** | **0.000** | NR_038975 | ref\|Homo sapiens MIR181A2 host gene (non-protein coding) (MIR181A2HG), long non-coding RNA [NR_038975] |
| 41 | DACT1 | **4.065** | **0.000** | NM_016651 | ref\|Homo sapiens dishevelled-binding antagonist of beta-catenin 1 (DACT1), transcript variant 1, mRNA [NM_016651] |
| 42 | FRMD5 | **4.052** | **0.013** | NM_001286491 | ref\|Homo sapiens FERM domain containing 5 (FRMD5), transcript variant 4, mRNA [NM_001286491] |
| 43 | SYT12 | **3.911** | **0.000** | NM_177963 | ref\|Homo sapiens synaptotagmin XII (SYT12), transcript variant 1, mRNA [NM_177963] |
| 44 | HTRA1 | **3.867** | **0.000** | NM_002775 | ref\|Homo sapiens HtrA serine peptidase 1 (HTRA1), mRNA [NM_002775] |
| 45 | HTR1D | **3.835** | **0.007** | NM_000864 | ref\|Homo sapiens 5-hydroxytryptamine (serotonin) receptor 1D, G protein-coupled (HTR1D), mRNA [NM_000864] |
| 46 | AMIGO2 | **3.734** | **0.000** | NM_181847 | ref\|Homo sapiens adhesion molecule with Ig-like domain 2 (AMIGO2), transcript variant 2, mRNA [NM_181847] |
| 47 | LDLRAD4 | **3.718** | **0.000** | NM_181482 | ref\|Homo sapiens low density lipoprotein receptor class A domain containing 4 (LDLRAD4), transcript variant a2, mRNA [NM_181482] |
| 48 | TYRP1 | **3.707** | **0.000** | NM_000550 | ref\|Homo sapiens tyrosinase-related protein 1 (TYRP1), mRNA [NM_000550] |
| 49 | ENST00000407780 | **3.674** | **0.000** | ENST00000407780 | ens\|inducible T-cell co-stimulator ligand [Source:HGNC Symbol;Acc:HGNC:17087] [ENST00000407780] |
| 50 | FHOD3 | **3.595** | **0.004** | NM_025135 | ref\|Homo sapiens formin homology 2 domain containing 3 (FHOD3), transcript variant 1, mRNA [NM_025135] |
| 51 | BPGM | **3.568** | **0.000** | NM_199186 | ref\|Homo sapiens 2,3-bisphosphoglycerate mutase (BPGM), transcript variant 2, mRNA [NM_199186] |
| 52 | CARD14 | **3.525** | **0.000** | NM_024110 | ref\|Homo sapiens caspase recruitment domain family, member 14 (CARD14), transcript variant 1, mRNA [NM_024110] |
| 53 | SKIL | **3.518** | **0.000** | NM_005414 | ref\|Homo sapiens SKI-like proto-oncogene (SKIL), transcript variant 1, mRNA [NM_005414] |
| 54 | SMAD7 | **3.460** | **0.000** | NM_005904 | ref\|Homo sapiens SMAD family member 7 (SMAD7), transcript variant 1, mRNA [NM_005904] |
| 55 | ZNF365 | **3.447** | **0.023** | NM_199451 | ref\|Homo sapiens zinc finger protein 365 (ZNF365), transcript variant C, mRNA [NM_199451] |
| 56 | NRP2 | **3.433** | **0.000** | NM_201264 | ref\|Homo sapiens neuropilin 2 (NRP2), transcript variant 6, mRNA [NM_201264] |
| 57 | ATP2B2 | **3.416** | **0.001** | NM_001001331 | ref\|Homo sapiens ATPase, Ca++ transporting, plasma membrane 2 (ATP2B2), transcript variant 1, mRNA [NM_001001331] |
| 58 | CALHM3 | **3.323** | **0.000** | NM_001129742 | ref\|Homo sapiens calcium homeostasis modulator 3 (CALHM3), mRNA [NM_001129742] |
| 59 | SAMD11 | **3.295** | **0.000** | NM_152486 | ref\|Homo sapiens sterile alpha motif domain containing 11 (SAMD11), mRNA [NM_152486] |
| 60 | ELSPBP1 | **3.256** | **0.000** | NM_022142 | ref\|Homo sapiens epididymal sperm binding protein 1 (ELSPBP1), mRNA [NM_022142] |
| 61 | PRICKLE1 | **3.232** | **0.002** | NM_153026 | ref\|Homo sapiens prickle homolog 1 (Drosophila) (PRICKLE1), transcript variant 1, mRNA [NM_153026] |
| 62 | CSF1R | **3.209** | **0.000** | NM_005211 | ref\|Homo sapiens colony stimulating factor 1 receptor (CSF1R), transcript variant 1, mRNA [NM_005211] |
| 63 | IHH | **3.059** | **0.002** | NM_002181 | ref\|Homo sapiens indian hedgehog (IHH), mRNA [NM_002181] |
| 64 | CD82 | **3.042** | **0.000** | NM_002231 | ref\|Homo sapiens CD82 molecule (CD82), transcript variant 1, mRNA [NM_002231] |
| 65 | NKD1 | **3.004** | **0.001** | NM_033119 | ref\|Homo sapiens naked cuticle homolog 1 (Drosophila) (NKD1), mRNA [NM_033119] |
| 66 | PRDM1 | **2.941** | **0.000** | NM_001198 | ref\|Homo sapiens PR domain containing 1, with ZNF domain (PRDM1), transcript variant 1, mRNA [NM_001198] |
| 67 | PBXIP1 | **2.912** | **0.000** | NM_020524 | ref\|Homo sapiens pre-B-cell leukemia homeobox interacting protein 1 (PBXIP1), mRNA [NM_020524] |
| 68 | CTGF | **2.899** | **0.000** | NM_001901 | ref\|Homo sapiens connective tissue growth factor (CTGF), mRNA [NM_001901] |
| 69 | DNM3 | **2.896** | **0.001** | NM_015569 | ref\|Homo sapiens dynamin 3 (DNM3), transcript variant 1, mRNA [NM_015569] |
| 70 | DAAM1 | **2.883** | **0.000** | NM_014992 | ref\|Homo sapiens dishevelled associated activator of morphogenesis 1 (DAAM1), transcript variant 1, mRNA [NM_014992] |
| 71 | SYTL2 | **2.821** | **0.000** | NM_032943 | ref\|Homo sapiens synaptotagmin-like 2 (SYTL2), transcript variant a, mRNA [NM_032943] |
| 72 | CEMIP | **2.804** | **0.011** | NM_018689 | ref\|Homo sapiens cell migration inducing protein, hyaluronan binding (CEMIP), transcript variant 3, mRNA [NM_018689] |
| 73 | SPOCD1 | **2.801** | **0.000** | NM_144569 | ref\|Homo sapiens SPOC domain containing 1 (SPOCD1), transcript variant 1, mRNA [NM_144569] |
| 74 | HMGA2 | **2.786** | **0.001** | NM_003483 | ref\|Homo sapiens high mobility group AT-hook 2 (HMGA2), transcript variant 1, mRNA [NM_003483] |
| 75 | ITGB3 | **2.728** | **0.000** | NM_000212 | ref\|Homo sapiens integrin, beta 3 (platelet glycoprotein IIIa, antigen CD61) (ITGB3), mRNA [NM_000212] |
| 76 | CTHRC1 | **2.709** | **0.000** | NM_138455 | ref\|Homo sapiens collagen triple helix repeat containing 1 (CTHRC1), transcript variant 1, mRNA [NM_138455] |
| 77 | TMIE | **2.707** | **0.016** | NM_147196 | ref\|Homo sapiens transmembrane inner ear (TMIE), mRNA [NM_147196] |
| 78 | FGF1 | **2.688** | **0.010** | NM_000800 | ref\|Homo sapiens fibroblast growth factor 1 (acidic) (FGF1), transcript variant 1, mRNA [NM_000800] |
| 79 | LMCD1 | **2.657** | **0.000** | NM_014583 | ref\|Homo sapiens LIM and cysteine-rich domains 1 (LMCD1), transcript variant 1, mRNA [NM_014583] |
| 80 | CRLF1 | **2.645** | **0.000** | NM_004750 | ref\|Homo sapiens cytokine receptor-like factor 1 (CRLF1), mRNA [NM_004750] |
| 81 | PRRX2 | **2.621** | **0.004** | NM_016307 | ref\|Homo sapiens paired related homeobox 2 (PRRX2), mRNA [NM_016307] |
| 82 | THC2755556 | **2.604** | **0.001** | THC2755556 | tc\|Q5T511_HUMAN (Q5T511) Leucine-rich repeat-containing G protein-coupled receptor 6 (Fragment), complete [THC2755556] |
| 83 | KCNK6 | **2.592** | **0.023** | NM_004823 | ref\|Homo sapiens potassium channel, two pore domain subfamily K, member 6 (KCNK6), mRNA [NM_004823] |
| 84 | ZNF423 | **2.584** | **0.008** | NM_015069 | ref\|Homo sapiens zinc finger protein 423 (ZNF423), transcript variant 1, mRNA [NM_015069] |
| 85 | PFKFB3 | **2.576** | **0.000** | NM_004566 | ref\|Homo sapiens 6-phosphofructo-2-kinase/fructose-2,6-biphosphatase 3 (PFKFB3), transcript variant 1, mRNA [NM_004566] |
| 86 | NDP | **2.556** | **0.009** | NM_000266 | ref\|Homo sapiens Norrie disease (pseudoglioma) (NDP), mRNA [NM_000266] |
| 87 | SORCS2 | **2.553** | **0.000** | NM_020777 | ref\|Homo sapiens sortilin-related VPS10 domain containing receptor 2 (SORCS2), mRNA [NM_020777] |
| 88 | SIK1 | **2.553** | **0.000** | NM_173354 | ref\|Homo sapiens salt-inducible kinase 1 (SIK1), mRNA [NM_173354] |
| 89 | CAMK4 | **2.533** | **0.002** | NM_001744 | ref\|Homo sapiens calcium/calmodulin-dependent protein kinase IV (CAMK4), mRNA [NM_001744] |
| 90 | TGFB1 | **2.528** | **0.002** | NM_000660 | ref\|Homo sapiens transforming growth factor, beta 1 (TGFB1), mRNA [NM_000660] |
| 91 | PLAT | **2.512** | **0.000** | NM_000930 | ref\|Homo sapiens plasminogen activator, tissue (PLAT), transcript variant 1, mRNA [NM_000930] |
| 92 | XKR7 | **2.483** | **0.042** | NM_001011718 | ref\|Homo sapiens XK, Kell blood group complex subunit-related family, member 7 (XKR7), mRNA [NM_001011718] |
| 93 | STEAP3 | **2.465** | **0.000** | NM_182915 | ref\|Homo sapiens STEAP family member 3, metalloreductase (STEAP3), transcript variant 1, mRNA [NM_182915] |
| 94 | FST | **2.426** | **0.000** | NM_013409 | ref\|Homo sapiens follistatin (FST), transcript variant FST344, mRNA [NM_013409] |
| 95 | PPAPDC1A | **2.423** | **0.001** | NM_001030059 | ref\|Homo sapiens phosphatidic acid phosphatase type 2 domain containing 1A (PPAPDC1A), mRNA [NM_001030059] |
| 96 | DRAXIN | **2.420** | **0.001** | NM_198545 | ref\|Homo sapiens dorsal inhibitory axon guidance protein (DRAXIN), mRNA [NM_198545] |
| 97 | ENC1 | **2.394** | **0.000** | NM_003633 | ref\|Homo sapiens ectodermal-neural cortex 1 (with BTB domain) (ENC1), transcript variant 1, mRNA [NM_003633] |
| 98 | ID1 | **2.387** | **0.000** | NM_002165 | ref\|Homo sapiens inhibitor of DNA binding 1, dominant negative helix-loop-helix protein (ID1), transcript variant 1, mRNA [NM_002165] |
| 99 | ADO | **2.375** | **0.018** | NM_032804 | ref\|Homo sapiens 2-aminoethanethiol (cysteamine) dioxygenase (ADO), mRNA [NM_032804] |
| 100 | NAV1 | **2.374** | **0.000** | NM_020443 | ref\|Homo sapiens neuron navigator 1 (NAV1), transcript variant 1, mRNA [NM_020443] |
| 101 | ATP1B1 | **2.374** | **0.000** | NM_001677 | ref\|Homo sapiens ATPase, Na+/K+ transporting, beta 1 polypeptide (ATP1B1), mRNA [NM_001677] |
| 102 | CDKN2B | **2.371** | **0.000** | NM_004936 | ref\|Homo sapiens cyclin-dependent kinase inhibitor 2B (p15, inhibits CDK4) (CDKN2B), transcript variant 1, mRNA [NM_004936] |
| 103 | VCAN | **2.340** | **0.000** | NM_004385 | ref\|Homo sapiens versican (VCAN), transcript variant 1, mRNA [NM_004385] |
| 104 | MEF2C | **2.333** | **0.001** | NM_002397 | ref\|Homo sapiens myocyte enhancer factor 2C (MEF2C), transcript variant 1, mRNA [NM_002397] |
| 105 | SPSB1 | **2.328** | **0.000** | NM_025106 | ref\|Homo sapiens splA/ryanodine receptor domain and SOCS box containing 1 (SPSB1), mRNA [NM_025106] |
| 106 | HILS1 | **2.321** | **0.009** | NR_024193 | ref\|Homo sapiens histone linker H1 domain, spermatid-specific 1, pseudogene (HILS1), transcript variant 1, non-coding RNA [NR_024193] |
| 107 | ANKH | **2.317** | **0.000** | NM_054027 | ref\|Homo sapiens ANKH inorganic pyrophosphate transport regulator (ANKH), mRNA [NM_054027] |
| 108 | LPL | **2.300** | **0.032** | NM_000237 | ref\|Homo sapiens lipoprotein lipase (LPL), mRNA [NM_000237] |
| 109 | TMCC2 | **2.296** | **0.001** | NM_014858 | ref\|Homo sapiens transmembrane and coiled-coil domain family 2 (TMCC2), transcript variant 1, mRNA [NM_014858] |
| 110 | TNFRSF12A | **2.281** | **0.004** | NM_016639 | ref\|Homo sapiens tumor necrosis factor receptor superfamily, member 12A (TNFRSF12A), mRNA [NM_016639] |
| 111 | PIK3IP1 | **2.271** | **0.037** | NM_052880 | ref\|Homo sapiens phosphoinositide-3-kinase interacting protein 1 (PIK3IP1), transcript variant 1, mRNA [NM_052880] |
| 112 | FGF18 | **2.257** | **0.002** | NM_003862 | ref\|Homo sapiens fibroblast growth factor 18 (FGF18), mRNA [NM_003862] |
| 113 | NREP | **2.230** | **0.000** | NM_001142483 | ref\|Homo sapiens neuronal regeneration related protein (NREP), transcript variant 11, mRNA [NM_001142483] |
| 114 | KCNMB4 | **2.229** | **0.000** | NM_014505 | ref\|Homo sapiens potassium channel subfamily M regulatory beta subunit 4 (KCNMB4), mRNA [NM_014505] |
| 115 | HECW2 | **2.222** | **0.001** | NM_020760 | ref\|Homo sapiens HECT, C2 and WW domain containing E3 ubiquitin protein ligase 2 (HECW2), mRNA [NM_020760] |
| 116 | XYLT1 | **2.219** | **0.030** | NM_022166 | ref\|Homo sapiens xylosyltransferase I (XYLT1), mRNA [NM_022166] |
| 117 | GAD1 | **2.218** | **0.005** | NM_000817 | ref\|Homo sapiens glutamate decarboxylase 1 (brain, 67kDa) (GAD1), transcript variant GAD67, mRNA [NM_000817] |
| 118 | RAMP1 | **2.215** | **0.000** | NM_005855 | ref\|Homo sapiens receptor (G protein-coupled) activity modifying protein 1 (RAMP1), mRNA [NM_005855] |
| 119 | SPINT2 | **2.197** | **0.003** | NM_021102 | ref\|Homo sapiens serine peptidase inhibitor, Kunitz type, 2 (SPINT2), transcript variant a, mRNA [NM_021102] |
| 120 | PTGDR | **2.193** | **0.032** | NM_000953 | ref\|Homo sapiens prostaglandin D2 receptor (DP) (PTGDR), transcript variant 1, mRNA [NM_000953] |
| 121 | TPM1 | **2.188** | **0.000** | NM_001018005 | ref\|Homo sapiens tropomyosin 1 (alpha) (TPM1), transcript variant Tpm1.1, mRNA [NM_001018005] |
| 122 | KLF7 | **2.185** | **0.001** | NM_003709 | ref\|Homo sapiens Kruppel-like factor 7 (ubiquitous) (KLF7), transcript variant 1, mRNA [NM_003709] |
| 123 | IL7R | **2.184** | **0.001** | NM_002185 | ref\|Homo sapiens interleukin 7 receptor (IL7R), transcript variant 1, mRNA [NM_002185] |
| 124 | LRIG3 | **2.169** | **0.000** | NM_153377 | ref\|Homo sapiens leucine-rich repeats and immunoglobulin-like domains 3 (LRIG3), transcript variant 2, mRNA [NM_153377] |
| 125 | EYA2 | **2.161** | **0.000** | NM_005244 | ref\|Homo sapiens EYA transcriptional coactivator and phosphatase 2 (EYA2), transcript variant 1, mRNA [NM_005244] |
| 126 | KIF26B | **2.159** | **0.000** | NM_018012 | ref\|Homo sapiens kinesin family member 26B (KIF26B), mRNA [NM_018012] |
| 127 | MFAP4 | **2.152** | **0.002** | NM_002404 | ref\|Homo sapiens microfibrillar-associated protein 4 (MFAP4), transcript variant 2, mRNA [NM_002404] |
| 128 | MAP3K4 | **2.149** | **0.000** | NM_005922 | ref\|Homo sapiens mitogen-activated protein kinase kinase kinase 4 (MAP3K4), transcript variant 1, mRNA [NM_005922] |
| 129 | ATP13A2 | **2.143** | **0.001** | NM_022089 | ref\|Homo sapiens ATPase type 13A2 (ATP13A2), transcript variant 1, mRNA [NM_022089] |
| 130 | SIGLEC17P | **2.141** | **0.000** | ENST00000611992 | ens\|sialic acid binding Ig-like lectin 17, pseudogene [Source:HGNC Symbol;Acc:HGNC:15604] [ENST00000611992] |
| 131 | PTPRR | **2.133** | **0.029** | NM_002849 | ref\|Homo sapiens protein tyrosine phosphatase, receptor type, R (PTPRR), transcript variant 1, mRNA [NM_002849] |
| 132 | ADAM19 | **2.117** | **0.001** | NM_033274 | ref\|Homo sapiens ADAM metallopeptidase domain 19 (ADAM19), mRNA [NM_033274] |
| 133 | TNFSF4 | **2.112** | **0.025** | NM_003326 | ref\|Homo sapiens tumor necrosis factor (ligand) superfamily, member 4 (TNFSF4), transcript variant 1, mRNA [NM_003326] |
| 134 | FAM26E | **2.083** | **0.000** | NM_153711 | ref\|Homo sapiens family with sequence similarity 26, member E (FAM26E), mRNA [NM_153711] |
| 135 | IFITM10 | **2.071** | **0.000** | NM_001170820 | ref\|Homo sapiens interferon induced transmembrane protein 10 (IFITM10), mRNA [NM_001170820] |
| 136 | ESP33 | **2.058** | **0.048** | XR_250562 | ref\|PREDICTED: Homo sapiens uncharacterized locus ESP33 (ESP33), misc_RNA [XR_250562] |
| 137 | EMP1 | **2.053** | **0.001** | NM_001423 | ref\|Homo sapiens epithelial membrane protein 1 (EMP1), mRNA [NM_001423] |
| 138 | MDFI | **2.050** | **0.003** | NM_001300804 | ref\|Homo sapiens MyoD family inhibitor (MDFI), transcript variant 1, mRNA [NM_001300804] |
| 139 | COL7A1 | **2.049** | **0.001** | NM_000094 | ref\|Homo sapiens collagen, type VII, alpha 1 (COL7A1), mRNA [NM_000094] |
| 140 | KLF10 | **2.046** | **0.002** | NM_005655 | ref\|Homo sapiens Kruppel-like factor 10 (KLF10), transcript variant 1, mRNA [NM_005655] |
| 141 | GEM | **2.043** | **0.001** | NM_005261 | ref\|Homo sapiens GTP binding protein overexpressed in skeletal muscle (GEM), transcript variant 1, mRNA [NM_005261] |
| 142 | COL4A1 | **2.033** | **0.002** | NM_001845 | ref\|Homo sapiens collagen, type IV, alpha 1 (COL4A1), transcript variant 1, mRNA [NM_001845] |
| 143 | BHLHE40 | **2.029** | **0.002** | NM_003670 | ref\|Homo sapiens basic helix-loop-helix family, member e40 (BHLHE40), mRNA [NM_003670] |
| 144 | S1PR3 | **2.027** | **0.000** | NM_005226 | ref\|Homo sapiens sphingosine-1-phosphate receptor 3 (S1PR3), mRNA [NM_005226] |
| 145 | CYR61 | **2.018** | **0.001** | NM_001554 | ref\|Homo sapiens cysteine-rich, angiogenic inducer, 61 (CYR61), mRNA [NM_001554] |
| 146 | DHRS3 | **2.017** | **0.006** | NM_004753 | ref\|Homo sapiens dehydrogenase/reductase (SDR family) member 3 (DHRS3), mRNA [NM_004753] |
| 147 | LPCAT2 | **2.015** | **0.002** | NM_017839 | ref\|Homo sapiens lysophosphatidylcholine acyltransferase 2 (LPCAT2), mRNA [NM_017839] |
| 148 | ARHGEF4 | **2.000** | **0.004** | NM_015320 | ref\|Homo sapiens Rho guanine nucleotide exchange factor (GEF) 4 (ARHGEF4), transcript variant 1, mRNA [NM_015320] |
| 149 | FZD8 | **1.992** | **0.001** | NM_031866 | ref\|Homo sapiens frizzled class receptor 8 (FZD8), mRNA [NM_031866] |
| 150 | HEY2 | **1.980** | **0.000** | NM_012259 | ref\|Homo sapiens hes-related family bHLH transcription factor with YRPW motif 2 (HEY2), mRNA [NM_012259] |
| 151 | PTHLH | **1.965** | **0.017** | NM_198965 | ref\|Homo sapiens parathyroid hormone-like hormone (PTHLH), transcript variant 1, mRNA [NM_198965] |
| 152 | USP18 | **1.961** | **0.000** | NM_017414 | ref\|Homo sapiens ubiquitin specific peptidase 18 (USP18), mRNA [NM_017414] |
| 153 | HLA-DOB | **1.951** | **0.027** | NM_002120 | ref\|Homo sapiens major histocompatibility complex, class II, DO beta (HLA-DOB), mRNA [NM_002120] |
| 154 | THBS1 | **1.948** | **0.000** | NM_003246 | ref\|Homo sapiens thrombospondin 1 (THBS1), mRNA [NM_003246] |
| 155 | F2R | **1.936** | **0.000** | BC016059 | gb\|Homo sapiens coagulation factor II (thrombin) receptor, mRNA (cDNA clone IMAGE:4849569), with apparent retained intron. [BC016059] |
| 156 | PHACTR1 | **1.929** | **0.004** | ENST00000379350 | ens\|phosphatase and actin regulator 1 [Source:HGNC Symbol;Acc:HGNC:20990] [ENST00000379350] |
| 157 | CDH6 | **1.927** | **0.001** | ENST00000506396 | ens\|cadherin 6, type 2, K-cadherin (fetal kidney) [Source:HGNC Symbol;Acc:HGNC:1765] [ENST00000506396] |
| 158 | HTR7 | **1.926** | **0.001** | NM_019859 | ref\|Homo sapiens 5-hydroxytryptamine (serotonin) receptor 7, adenylate cyclase-coupled (HTR7), transcript variant d, mRNA [NM_019859] |
| 159 | DLX2 | **1.924** | **0.000** | NM_004405 | ref\|Homo sapiens distal-less homeobox 2 (DLX2), mRNA [NM_004405] |
| 160 | B3GNT5 | **1.922** | **0.002** | NM_032047 | ref\|Homo sapiens UDP-GlcNAc:betaGal beta-1,3-N-acetylglucosaminyltransferase 5 (B3GNT5), mRNA [NM_032047] |
| 161 | DLC1 | **1.915** | **0.000** | NM_024767 | ref\|Homo sapiens DLC1 Rho GTPase activating protein (DLC1), transcript variant 3, mRNA [NM_024767] |
| 162 | LOXL4 | **1.913** | **0.024** | NM_032211 | ref\|Homo sapiens lysyl oxidase-like 4 (LOXL4), mRNA [NM_032211] |
| 163 | CACNA1C | **1.906** | **0.001** | NM_199460 | ref\|Homo sapiens calcium channel, voltage-dependent, L type, alpha 1C subunit (CACNA1C), transcript variant 1, mRNA [NM_199460] |
| 164 | LFNG | **1.895** | **0.005** | NM_001040168 | ref\|Homo sapiens LFNG O-fucosylpeptide 3-beta-N-acetylglucosaminyltransferase (LFNG), transcript variant 2, mRNA [NM_001040168] |
| 165 | F2RL2 | **1.885** | **0.001** | NM_004101 | ref\|Homo sapiens coagulation factor II (thrombin) receptor-like 2 (F2RL2), transcript variant 1, mRNA [NM_004101] |
| 166 | BMF | **1.880** | **0.001** | NM_001003940 | ref\|Homo sapiens Bcl2 modifying factor (BMF), transcript variant 1, mRNA [NM_001003940] |
| 167 | LRRC17 | **1.878** | **0.001** | NM_005824 | ref\|Homo sapiens leucine rich repeat containing 17 (LRRC17), transcript variant 2, mRNA [NM_005824] |
| 168 | EFR3B | **1.872** | **0.003** | NM_014971 | ref\|Homo sapiens EFR3 homolog B (S. cerevisiae) (EFR3B), mRNA [NM_014971] |
| 169 | PCED1B | **1.871** | **0.012** | NM_138371 | ref\|Homo sapiens PC-esterase domain containing 1B (PCED1B), transcript variant 1, mRNA [NM_138371] |
| 170 | PPP1R3B | **1.861** | **0.001** | NM_001201329 | ref\|Homo sapiens protein phosphatase 1, regulatory subunit 3B (PPP1R3B), transcript variant 1, mRNA [NM_001201329] |
| 171 | CMTM1 | **1.856** | **0.012** | NM_052999 | ref\|Homo sapiens CKLF-like MARVEL transmembrane domain containing 1 (CMTM1), transcript variant 17, mRNA [NM_052999] |
| 172 | TLE3 | **1.853** | **0.000** | NM_005078 | ref\|Homo sapiens transducin-like enhancer of split 3 (TLE3), transcript variant 1, mRNA [NM_005078] |
| 173 | AGPS | **1.850** | **0.000** | NM_003659 | ref\|Homo sapiens alkylglycerone phosphate synthase (AGPS), mRNA [NM_003659] |
| 174 | ACSL1 | **1.849** | **0.002** | NM_001995 | ref\|Homo sapiens acyl-CoA synthetase long-chain family member 1 (ACSL1), transcript variant 1, mRNA [NM_001995] |
| 175 | HBEGF | **1.848** | **0.002** | NM_001945 | ref\|Homo sapiens heparin-binding EGF-like growth factor (HBEGF), mRNA [NM_001945] |
| 176 | RHOBTB1 | **1.847** | **0.003** | NM_001242359 | ref\|Homo sapiens Rho-related BTB domain containing 1 (RHOBTB1), transcript variant 4, mRNA [NM_001242359] |
| 177 | LMO1 | **1.847** | **0.013** | NM_002315 | ref\|Homo sapiens LIM domain only 1 (rhombotin 1) (LMO1), transcript variant 1, mRNA [NM_002315] |
| 178 | VDR | **1.843** | **0.000** | NM_001017535 | ref\|Homo sapiens vitamin D (1,25- dihydroxyvitamin D3) receptor (VDR), transcript variant 2, mRNA [NM_001017535] |
| 179 | KIAA1324 | **1.840** | **0.000** | NM_020775 | ref\|Homo sapiens KIAA1324 (KIAA1324), transcript variant 1, mRNA [NM_020775] |
| 180 | PRR16 | **1.839** | **0.033** | NM_016644 | ref\|Homo sapiens proline rich 16 (PRR16), transcript variant 2, mRNA [NM_016644] |
| 181 | CNIH3 | **1.839** | **0.002** | NM_152495 | ref\|Homo sapiens cornichon family AMPA receptor auxiliary protein 3 (CNIH3), mRNA [NM_152495] |
| 182 | SUSD6 | **1.831** | **0.001** | NM_014734 | ref\|Homo sapiens sushi domain containing 6 (SUSD6), mRNA [NM_014734] |
| 183 | GALNT10 | **1.816** | **0.000** | NM_198321 | ref\|Homo sapiens polypeptide N-acetylgalactosaminyltransferase 10 (GALNT10), mRNA [NM_198321] |
| 184 | PXDC1 | **1.811** | **0.000** | NM_183373 | ref\|Homo sapiens PX domain containing 1 (PXDC1), mRNA [NM_183373] |
| 185 | MAF | **1.810** | **0.003** | NM_001031804 | ref\|Homo sapiens v-maf avian musculoaponeurotic fibrosarcoma oncogene homolog (MAF), transcript variant 2, mRNA [NM_001031804] |
| 186 | MAPRE3 | **1.809** | **0.002** | NM_012326 | ref\|Homo sapiens microtubule-associated protein, RP/EB family, member 3 (MAPRE3), transcript variant 1, mRNA [NM_012326] |
| 187 | LIMS3L | **1.807** | **0.012** | NR_038099 | ref\|Homo sapiens LIM and senescent cell antigen-like domains 3-like (LIMS3L), transcript variant 2, non-coding RNA [NR_038099] |
| 188 | HIC1 | **1.805** | **0.006** | NM_006497 | ref\|Homo sapiens hypermethylated in cancer 1 (HIC1), transcript variant 1, mRNA [NM_006497] |
| 189 | SH3BP4 | **1.786** | **0.002** | NM_014521 | ref\|Homo sapiens SH3-domain binding protein 4 (SH3BP4), mRNA [NM_014521] |
| 190 | UCN2 | **1.774** | **0.000** | NM_033199 | ref\|Homo sapiens urocortin 2 (UCN2), mRNA [NM_033199] |
| 191 | IL1RAP | **1.773** | **0.008** | NM_134470 | ref\|Homo sapiens interleukin 1 receptor accessory protein (IL1RAP), transcript variant 2, mRNA [NM_134470] |
| 192 | SGK1 | **1.772** | **0.001** | NM_005627 | ref\|Homo sapiens serum/glucocorticoid regulated kinase 1 (SGK1), transcript variant 1, mRNA [NM_005627] |
| 193 | DOCK3 | **1.768** | **0.002** | NM_004947 | ref\|Homo sapiens dedicator of cytokinesis 3 (DOCK3), mRNA [NM_004947] |
| 194 | SLC24A3 | **1.760** | **0.002** | NM_020689 | ref\|Homo sapiens solute carrier family 24 (sodium/potassium/calcium exchanger), member 3 (SLC24A3), mRNA [NM_020689] |
| 195 | GFRA2 | **1.753** | **0.000** | NM_001495 | ref\|Homo sapiens GDNF family receptor alpha 2 (GFRA2), transcript variant 1, mRNA [NM_001495] |
| 196 | ZNF827 | **1.752** | **0.012** | NM_178835 | ref\|Homo sapiens zinc finger protein 827 (ZNF827), mRNA [NM_178835] |
| 197 | PHLDB1 | **1.747** | **0.001** | NM_015157 | ref\|Homo sapiens pleckstrin homology-like domain, family B, member 1 (PHLDB1), transcript variant 1, mRNA [NM_015157] |
| 198 | OSBPL10 | **1.745** | **0.001** | NM_017784 | ref\|Homo sapiens oxysterol binding protein-like 10 (OSBPL10), transcript variant 1, mRNA [NM_017784] |
| 199 | TCAF2 | **1.739** | **0.003** | NM_001130026 | ref\|Homo sapiens family with sequence similarity 115, member C (FAM115C), transcript variant 3, mRNA [NM_001130026] |
| 200 | HEY1 | **1.738** | **0.000** | NM_001040708 | ref\|Homo sapiens hes-related family bHLH transcription factor with YRPW motif 1 (HEY1), transcript variant 2, mRNA [NM_001040708] |
| 201 | SH2B3 | **1.738** | **0.001** | NM_005475 | ref\|Homo sapiens SH2B adaptor protein 3 (SH2B3), transcript variant 1, mRNA [NM_005475] |
| 202 | SKI | **1.737** | **0.002** | NM_003036 | ref\|Homo sapiens v-ski avian sarcoma viral oncogene homolog (SKI), mRNA [NM_003036] |
| 203 | GPR183 | **1.734** | **0.002** | NM_004951 | ref\|Homo sapiens G protein-coupled receptor 183 (GPR183), mRNA [NM_004951] |
| 204 | ZNF521 | **1.730** | **0.001** | NM_015461 | ref\|Homo sapiens zinc finger protein 521 (ZNF521), mRNA [NM_015461] |
| 205 | GFPT2 | **1.728** | **0.000** | NM_005110 | ref\|Homo sapiens glutamine-fructose-6-phosphate transaminase 2 (GFPT2), mRNA [NM_005110] |
| 206 | SMURF2 | **1.717** | **0.009** | NM_022739 | ref\|Homo sapiens SMAD specific E3 ubiquitin protein ligase 2 (SMURF2), mRNA [NM_022739] |
| 207 | SGCA | **1.713** | **0.003** | NM_000023 | ref\|Homo sapiens sarcoglycan, alpha (50kDa dystrophin-associated glycoprotein) (SGCA), transcript variant 1, mRNA [NM_000023] |
| 208 | PODNL1 | **1.709** | **0.001** | NM_024825 | ref\|Homo sapiens podocan-like 1 (PODNL1), transcript variant 1, mRNA [NM_024825] |
| 209 | BHLHE41 | **1.708** | **0.001** | NM_030762 | ref\|Homo sapiens basic helix-loop-helix family, member e41 (BHLHE41), mRNA [NM_030762] |
| 210 | TBC1D10C | **1.696** | **0.005** | NM_198517 | ref\|Homo sapiens TBC1 domain family, member 10C (TBC1D10C), transcript variant 1, mRNA [NM_198517] |
| 211 | ARL5C | **1.680** | **0.031** | NM_001143968 | ref\|Homo sapiens ADP-ribosylation factor-like 5C (ARL5C), mRNA [NM_001143968] |
| 212 | EFR3A | **1.677** | **0.001** | NM_015137 | ref\|Homo sapiens EFR3 homolog A (S. cerevisiae) (EFR3A), mRNA [NM_015137] |
| 213 | EPHB2 | **1.673** | **0.000** | NM_004442 | ref\|Homo sapiens EPH receptor B2 (EPHB2), transcript variant 2, mRNA [NM_004442] |
| 214 | ATP9A | **1.668** | **0.000** | NM_006045 | ref\|Homo sapiens ATPase, class II, type 9A (ATP9A), mRNA [NM_006045] |
| 215 | CREB3L2 | **1.661** | **0.000** | NM_001253775 | ref\|Homo sapiens cAMP responsive element binding protein 3-like 2 (CREB3L2), transcript variant 2, mRNA [NM_001253775] |
| 216 | LIMS1 | **1.661** | **0.000** | NM_004987 | ref\|Homo sapiens LIM and senescent cell antigen-like domains 1 (LIMS1), transcript variant 2, mRNA [NM_004987] |
| 217 | PPP1R13L | **1.653** | **0.000** | NM_006663 | ref\|Homo sapiens protein phosphatase 1, regulatory subunit 13 like (PPP1R13L), transcript variant 2, mRNA [NM_006663] |
| 218 | GBP1 | **1.649** | **0.000** | NM_002053 | ref\|Homo sapiens guanylate binding protein 1, interferon-inducible (GBP1), mRNA [NM_002053] |
| 219 | SNAI1 | **1.649** | **0.000** | NM_005985 | ref\|Homo sapiens snail family zinc finger 1 (SNAI1), mRNA [NM_005985] |
| 220 | SAMD5 | **1.645** | **0.004** | NM_001030060 | ref\|Homo sapiens sterile alpha motif domain containing 5 (SAMD5), mRNA [NM_001030060] |
| 221 | GDNF | **1.638** | **0.001** | NM_001190468 | ref\|Homo sapiens glial cell derived neurotrophic factor (GDNF), transcript variant 3, mRNA [NM_001190468] |
| 222 | ITPRIP | **1.635** | **0.005** | NM_033397 | ref\|Homo sapiens inositol 1,4,5-trisphosphate receptor interacting protein (ITPRIP), transcript variant 1, mRNA [NM_033397] |
| 223 | KHDRBS3 | **1.611** | **0.001** | NM_006558 | ref\|Homo sapiens KH domain containing, RNA binding, signal transduction associated 3 (KHDRBS3), mRNA [NM_006558] |
| 224 | PTPRU | **1.606** | **0.000** | NM_005704 | ref\|Homo sapiens protein tyrosine phosphatase, receptor type, U (PTPRU), transcript variant 3, mRNA [NM_005704] |
| 225 | TRIB1 | **1.602** | **0.000** | NM_025195 | ref\|Homo sapiens tribbles pseudokinase 1 (TRIB1), transcript variant 1, mRNA [NM_025195] |
| 226 | SPON2 | **1.594** | **0.019** | NM_012445 | ref\|Homo sapiens spondin 2, extracellular matrix protein (SPON2), transcript variant 1, mRNA [NM_012445] |
| 227 | KCNN4 | **1.582** | **0.000** | NM_002250 | ref\|Homo sapiens potassium channel, calcium activated intermediate/small conductance subfamily N alpha, member 4 (KCNN4), mRNA [NM_002250] |
| 228 | FOSB | **1.575** | **0.013** | NM_006732 | ref\|Homo sapiens FBJ murine osteosarcoma viral oncogene homolog B (FOSB), transcript variant 1, mRNA [NM_006732] |
| 229 | NRBF2 | **1.541** | **0.000** | NM_030759 | ref\|Homo sapiens nuclear receptor binding factor 2 (NRBF2), transcript variant 1, mRNA [NM_030759] |
| 230 | HLF | **0.165** | **0.001** | NM_002126 | ref\|Homo sapiens hepatic leukemia factor (HLF), mRNA [NM_002126] |
| 231 | KCNB1 | **0.180** | **0.000** | ENST00000371741 | ens\|potassium voltage-gated channel, Shab-related subfamily, member 1 [Source:HGNC Symbol;Acc:HGNC:6231] [ENST00000371741] |
| 232 | FOXQ1 | **0.652** | **0.002** | NM_033260 | ref\|Homo sapiens forkhead box Q1 (FOXQ1), mRNA [NM_033260] |
| 233 | IMPA2 | **0.646** | **0.000** | NM_014214 | ref\|Homo sapiens inositol(myo)-1(or 4)-monophosphatase 2 (IMPA2), mRNA [NM_014214] |
| 234 | TBC1D8 | **0.644** | **0.001** | NM_001102426 | ref\|Homo sapiens TBC1 domain family, member 8 (with GRAM domain) (TBC1D8), mRNA [NM_001102426] |
| 235 | CABLES1 | **0.630** | **0.000** | NM_138375 | ref\|Homo sapiens Cdk5 and Abl enzyme substrate 1 (CABLES1), transcript variant 1, mRNA [NM_138375] |
| 236 | GCLC | **0.626** | **0.001** | NM_001498 | ref\|Homo sapiens glutamate-cysteine ligase, catalytic subunit (GCLC), transcript variant 1, mRNA [NM_001498] |
| 237 | LRP5 | **0.625** | **0.000** | NM_002335 | ref\|Homo sapiens low density lipoprotein receptor-related protein 5 (LRP5), transcript variant 1, mRNA [NM_002335] |
| 238 | NTN4 | **0.621** | **0.010** | NM_021229 | ref\|Homo sapiens netrin 4 (NTN4), mRNA [NM_021229] |
| 239 | IFIT1 | **0.620** | **0.001** | NM_001548 | ref\|Homo sapiens interferon-induced protein with tetratricopeptide repeats 1 (IFIT1), transcript variant 1, mRNA [NM_001548] |
| 240 | MINOS1-NBL1 | **0.619** | **0.000** | NM_001204088 | ref\|Homo sapiens MINOS1-NBL1 readthrough (MINOS1-NBL1), transcript variant 1, mRNA [NM_001204088] |
| 241 | NOV | **0.615** | **0.000** | NM_002514 | ref\|Homo sapiens nephroblastoma overexpressed (NOV), mRNA [NM_002514] |
| 242 | METTL7A | **0.614** | **0.000** | NM_014033 | ref\|Homo sapiens methyltransferase like 7A (METTL7A), mRNA [NM_014033] |
| 243 | ALDH3A1 | **0.613** | **0.000** | NM_000691 | ref\|Homo sapiens aldehyde dehydrogenase 3 family, member A1 (ALDH3A1), transcript variant 2, mRNA [NM_000691] |
| 244 | PPAP2B | **0.613** | **0.000** | NM_003713 | ref\|Homo sapiens phosphatidic acid phosphatase type 2B (PPAP2B), mRNA [NM_003713] |
| 245 | NEFH | **0.611** | **0.001** | NM_021076 | ref\|Homo sapiens neurofilament, heavy polypeptide (NEFH), mRNA [NM_021076] |
| 246 | C3 | **0.610** | **0.000** | NM_000064 | ref\|Homo sapiens complement component 3 (C3), mRNA [NM_000064] |
| 247 | ARIH2OS | **0.610** | **0.006** | NM_001123040 | ref\|Homo sapiens ariadne homolog 2 opposite strand (ARIH2OS), mRNA [NM_001123040] |
| 248 | IFIH1 | **0.609** | **0.000** | NM_022168 | ref\|Homo sapiens interferon induced with helicase C domain 1 (IFIH1), mRNA [NM_022168] |
| 249 | SLC47A2 | **0.609** | **0.004** | NM_152908 | ref\|Homo sapiens solute carrier family 47 (multidrug and toxin extrusion), member 2 (SLC47A2), transcript variant 1, mRNA [NM_152908] |
| 250 | PNPLA7 | **0.608** | **0.000** | NM_001098537 | ref\|Homo sapiens patatin-like phospholipase domain containing 7 (PNPLA7), transcript variant 1, mRNA [NM_001098537] |
| 251 | PDE5A | **0.606** | **0.000** | NM_001083 | ref\|Homo sapiens phosphodiesterase 5A, cGMP-specific (PDE5A), transcript variant 1, mRNA [NM_001083] |
| 252 | ALDH3A2 | **0.603** | **0.000** | NM_001031806 | ref\|Homo sapiens aldehyde dehydrogenase 3 family, member A2 (ALDH3A2), transcript variant 1, mRNA [NM_001031806] |
| 253 | KCNC1 | **0.602** | **0.001** | NM_004976 | ref\|Homo sapiens potassium channel, voltage gated Shaw related subfamily C, member 1 (KCNC1), transcript variant 2, mRNA [NM_004976] |
| 254 | RGCC | **0.599** | **0.000** | NM_014059 | ref\|Homo sapiens regulator of cell cycle (RGCC), mRNA [NM_014059] |
| 255 | OSGIN1 | **0.599** | **0.001** | NM_182981 | ref\|Homo sapiens oxidative stress induced growth inhibitor 1 (OSGIN1), mRNA [NM_182981] |
| 256 | LIMCH1 | **0.597** | **0.002** | NM_014988 | ref\|Homo sapiens LIM and calponin homology domains 1 (LIMCH1), transcript variant 1, mRNA [NM_014988] |
| 257 | SLC47A1 | **0.597** | **0.001** | NM_018242 | ref\|Homo sapiens solute carrier family 47 (multidrug and toxin extrusion), member 1 (SLC47A1), mRNA [NM_018242] |
| 258 | NRG2 | **0.595** | **0.001** | AK124504 | gb\|Homo sapiens cDNA FLJ42513 fis, clone BRACE2046295, highly similar to Pro-neuregulin-2, membrane-bound isoform precursor. [AK124504] |
| 259 | PTPRJ | **0.595** | **0.002** | NM_002843 | ref\|Homo sapiens protein tyrosine phosphatase, receptor type, J (PTPRJ), transcript variant 1, mRNA [NM_002843] |
| 260 | RAB26 | **0.594** | **0.000** | NM_014353 | ref\|Homo sapiens RAB26, member RAS oncogene family (RAB26), mRNA [NM_014353] |
| 261 | PLEKHA6 | **0.593** | **0.001** | NM_014935 | ref\|Homo sapiens pleckstrin homology domain containing, family A member 6 (PLEKHA6), mRNA [NM_014935] |
| 262 | MESP1 | **0.590** | **0.006** | NM_018670 | ref\|Homo sapiens mesoderm posterior basic helix-loop-helix transcription factor 1 (MESP1), mRNA [NM_018670] |
| 263 | STEAP1 | **0.590** | **0.005** | NM_012449 | ref\|Homo sapiens six transmembrane epithelial antigen of the prostate 1 (STEAP1), mRNA [NM_012449] |
| 264 | HIPK2 | **0.589** | **0.000** | NM_001113239 | ref\|Homo sapiens homeodomain interacting protein kinase 2 (HIPK2), transcript variant 2, mRNA [NM_001113239] |
| 265 | SIX1 | **0.584** | **0.000** | NM_005982 | ref\|Homo sapiens SIX homeobox 1 (SIX1), mRNA [NM_005982] |
| 266 | CMTM8 | **0.583** | **0.000** | NM_178868 | ref\|Homo sapiens CKLF-like MARVEL transmembrane domain containing 8 (CMTM8), mRNA [NM_178868] |
| 267 | ST3GAL1 | **0.582** | **0.001** | NM_003033 | ref\|Homo sapiens ST3 beta-galactoside alpha-2,3-sialyltransferase 1 (ST3GAL1), transcript variant 1, mRNA [NM_003033] |
| 268 | SLPI | **0.574** | **0.000** | NM_003064 | ref\|Homo sapiens secretory leukocyte peptidase inhibitor (SLPI), mRNA [NM_003064] |
| 269 | DAZL | **0.573** | **0.000** | NM_001351 | ref\|Homo sapiens deleted in azoospermia-like (DAZL), transcript variant 2, mRNA [NM_001351] |
| 270 | HPGD | **0.569** | **0.040** | NM_000860 | ref\|Homo sapiens hydroxyprostaglandin dehydrogenase 15-(NAD) (HPGD), transcript variant 1, mRNA [NM_000860] |
| 271 | STEAP2 | **0.568** | **0.001** | NM_001244944 | ref\|Homo sapiens STEAP family member 2, metalloreductase (STEAP2), transcript variant 4, mRNA [NM_001244944] |
| 272 | REP15 | **0.565** | **0.049** | NM_001029874 | ref\|Homo sapiens RAB15 effector protein (REP15), mRNA [NM_001029874] |
| 273 | INHBB | **0.564** | **0.001** | NM_002193 | ref\|Homo sapiens inhibin, beta B (INHBB), mRNA [NM_002193] |
| 274 | FAM117B | **0.563** | **0.001** | NM_173511 | ref\|Homo sapiens family with sequence similarity 117, member B (FAM117B), mRNA [NM_173511] |
| 275 | DEPTOR | **0.561** | **0.006** | NM_022783 | ref\|Homo sapiens DEP domain containing MTOR-interacting protein (DEPTOR), transcript variant 1, mRNA [NM_022783] |
| 276 | SLC12A7 | **0.561** | **0.000** | NM_006598 | ref\|Homo sapiens solute carrier family 12 (potassium/chloride transporter), member 7 (SLC12A7), mRNA [NM_006598] |
| 277 | EPHA4 | **0.559** | **0.000** | NM_004438 | ref\|Homo sapiens EPH receptor A4 (EPHA4), mRNA [NM_004438] |
| 278 | SOBP | **0.558** | **0.000** | NM_018013 | ref\|Homo sapiens sine oculis binding protein homolog (Drosophila) (SOBP), mRNA [NM_018013] |
| 279 | FMO3 | **0.557** | **0.003** | NM_001002294 | ref\|Homo sapiens flavin containing monooxygenase 3 (FMO3), transcript variant 2, mRNA [NM_001002294] |
| 280 | DRD2 | **0.556** | **0.000** | NM_000795 | ref\|Homo sapiens dopamine receptor D2 (DRD2), transcript variant 1, mRNA [NM_000795] |
| 281 | SOWAHD | **0.555** | **0.001** | NM_001105576 | ref\|Homo sapiens sosondowah ankyrin repeat domain family member D (SOWAHD), mRNA [NM_001105576] |
| 282 | GLCCI1 | **0.554** | **0.001** | NM_138426 | ref\|Homo sapiens glucocorticoid induced transcript 1 (GLCCI1), mRNA [NM_138426] |
| 283 | TMEM56 | **0.554** | **0.002** | NM_152487 | ref\|Homo sapiens transmembrane protein 56 (TMEM56), transcript variant 2, mRNA [NM_152487] |
| 284 | GUCY1A3 | **0.553** | **0.000** | NM_000856 | ref\|Homo sapiens guanylate cyclase 1, soluble, alpha 3 (GUCY1A3), transcript variant 1, mRNA [NM_000856] |
| 285 | PAMR1 | **0.553** | **0.000** | NM_015430 | ref\|Homo sapiens peptidase domain containing associated with muscle regeneration 1 (PAMR1), transcript variant 1, mRNA [NM_015430] |
| 286 | TLE1 | **0.552** | **0.000** | NM_001303104 | ref\|Homo sapiens transducin-like enhancer of split 1 (E(sp1) homolog, Drosophila) (TLE1), transcript variant 3, mRNA [NM_001303104] |
| 287 | ARL4D | **0.552** | **0.001** | NM_001661 | ref\|Homo sapiens ADP-ribosylation factor-like 4D (ARL4D), mRNA [NM_001661] |
| 288 | SEMA3C | **0.551** | **0.002** | NM_006379 | ref\|Homo sapiens sema domain, immunoglobulin domain (Ig), short basic domain, secreted, (semaphorin) 3C (SEMA3C), mRNA [NM_006379] |
| 289 | SEMA5A | **0.547** | **0.001** | NM_003966 | ref\|Homo sapiens sema domain, seven thrombospondin repeats (type 1 and type 1-like), transmembrane domain (TM) and short cytoplasmic domain, (semaphorin) 5A (SEMA5A), mRNA [NM_003966] |
| 290 | ERMP1 | **0.542** | **0.005** | NM_024896 | ref\|Homo sapiens endoplasmic reticulum metallopeptidase 1 (ERMP1), mRNA [NM_024896] |
| 291 | TMEM64 | **0.542** | **0.000** | NM_001008495 | ref\|Homo sapiens transmembrane protein 64 (TMEM64), transcript variant 1, mRNA [NM_001008495] |
| 292 | ARHGAP9 | **0.539** | **0.001** | NM_032496 | ref\|Homo sapiens Rho GTPase activating protein 9 (ARHGAP9), transcript variant 1, mRNA [NM_032496] |
| 293 | AF119870 | **0.537** | **0.006** | AF119870 | gb\|Homo sapiens PRO2266 mRNA, complete cds. [AF119870] |
| 294 | APOBEC3H | **0.532** | **0.006** | NM_181773 | ref\|Homo sapiens apolipoprotein B mRNA editing enzyme, catalytic polypeptide-like 3H (APOBEC3H), transcript variant SV-183, mRNA [NM_181773] |
| 295 | SLFNL1 | **0.532** | **0.001** | NM_144990 | ref\|Homo sapiens schlafen-like 1 (SLFNL1), transcript variant 1, mRNA [NM_144990] |
| 296 | CX3CL1 | **0.531** | **0.001** | ENST00000006053 | ens\|chemokine (C-X3-C motif) ligand 1 [Source:HGNC Symbol;Acc:HGNC:10647] [ENST00000006053] |
| 297 | FOXF1 | **0.531** | **0.000** | NM_001451 | ref\|Homo sapiens forkhead box F1 (FOXF1), mRNA [NM_001451] |
| 298 | LRIG1 | **0.530** | **0.001** | NM_015541 | ref\|Homo sapiens leucine-rich repeats and immunoglobulin-like domains 1 (LRIG1), mRNA [NM_015541] |
| 299 | ARHGAP20 | **0.528** | **0.000** | NM_020809 | ref\|Homo sapiens Rho GTPase activating protein 20 (ARHGAP20), transcript variant 1, mRNA [NM_020809] |
| 300 | BIRC3 | **0.527** | **0.005** | NM_001165 | ref\|Homo sapiens baculoviral IAP repeat containing 3 (BIRC3), transcript variant 1, mRNA [NM_001165] |
| 301 | PPL | **0.520** | **0.003** | NM_002705 | ref\|Homo sapiens periplakin (PPL), mRNA [NM_002705] |
| 302 | GHRLOS | **0.520** | **0.011** | NR_073566 | ref\|Homo sapiens ghrelin opposite strand/antisense RNA (GHRLOS), transcript variant 5, long non-coding RNA [NR_073566] |
| 303 | JPH1 | **0.518** | **0.010** | NM_020647 | ref\|Homo sapiens junctophilin 1 (JPH1), mRNA [NM_020647] |
| 304 | SLC51B | **0.515** | **0.009** | NM_178859 | ref\|Homo sapiens solute carrier family 51, beta subunit (SLC51B), mRNA [NM_178859] |
| 305 | FAM49A | **0.514** | **0.000** | NM_030797 | ref\|Homo sapiens family with sequence similarity 49, member A (FAM49A), mRNA [NM_030797] |
| 306 | MYBPH | **0.512** | **0.002** | NM_004997 | ref\|Homo sapiens myosin binding protein H (MYBPH), mRNA [NM_004997] |
| 307 | FLRT3 | **0.512** | **0.012** | NM_198391 | ref\|Homo sapiens fibronectin leucine rich transmembrane protein 3 (FLRT3), transcript variant 2, mRNA [NM_198391] |
| 308 | ADRB1 | **0.510** | **0.007** | NM_000684 | ref\|Homo sapiens adrenoceptor beta 1 (ADRB1), mRNA [NM_000684] |
| 309 | ADAMTS9 | **0.509** | **0.001** | NM_182920 | ref\|Homo sapiens ADAM metallopeptidase with thrombospondin type 1 motif, 9 (ADAMTS9), mRNA [NM_182920] |
| 310 | ITGA6 | **0.509** | **0.000** | NM_000210 | ref\|Homo sapiens integrin, alpha 6 (ITGA6), transcript variant 2, mRNA [NM_000210] |
| 311 | SLC12A2 | **0.503** | **0.008** | NM_001046 | ref\|Homo sapiens solute carrier family 12 (sodium/potassium/chloride transporter), member 2 (SLC12A2), transcript variant 1, mRNA [NM_001046] |
| 312 | EPAS1 | **0.501** | **0.000** | NM_001430 | ref\|Homo sapiens endothelial PAS domain protein 1 (EPAS1), mRNA [NM_001430] |
| 313 | ITGAM | **0.500** | **0.010** | NM_000632 | ref\|Homo sapiens integrin, alpha M (complement component 3 receptor 3 subunit) (ITGAM), transcript variant 2, mRNA [NM_000632] |
| 314 | MAP7D2 | **0.500** | **0.009** | NM_152780 | ref\|Homo sapiens MAP7 domain containing 2 (MAP7D2), transcript variant 2, mRNA [NM_152780] |
| 315 | OSR1 | **0.496** | **0.006** | NM_145260 | ref\|Homo sapiens odd-skipped related transciption factor 1 (OSR1), mRNA [NM_145260] |
| 316 | PPM1E | **0.496** | **0.027** | NM_014906 | ref\|Homo sapiens protein phosphatase, Mg2+/Mn2+ dependent, 1E (PPM1E), transcript variant 1, mRNA [NM_014906] |
| 317 | NR6A1 | **0.493** | **0.001** | NM_033334 | ref\|Homo sapiens nuclear receptor subfamily 6, group A, member 1 (NR6A1), transcript variant 1, mRNA [NM_033334] |
| 318 | SOCS1 | **0.492** | **0.001** | NM_003745 | ref\|Homo sapiens suppressor of cytokine signaling 1 (SOCS1), mRNA [NM_003745] |
| 319 | RORC | **0.490** | **0.018** | NM_005060 | ref\|Homo sapiens RAR-related orphan receptor C (RORC), transcript variant 1, mRNA [NM_005060] |
| 320 | KITLG | **0.489** | **0.030** | NM_000899 | ref\|Homo sapiens KIT ligand (KITLG), transcript variant b, mRNA [NM_000899] |
| 321 | SLIT2 | **0.488** | **0.000** | NM_004787 | ref\|Homo sapiens slit homolog 2 (Drosophila) (SLIT2), transcript variant 1, mRNA [NM_004787] |
| 322 | SCN4B | **0.486** | **0.007** | NM_174934 | ref\|Homo sapiens sodium channel, voltage gated, type IV beta subunit (SCN4B), transcript variant 1, mRNA [NM_174934] |
| 323 | CLMN | **0.483** | **0.003** | NM_024734 | ref\|Homo sapiens calmin (calponin-like, transmembrane) (CLMN), mRNA [NM_024734] |
| 324 | FAM110B | **0.482** | **0.000** | NM_147189 | ref\|Homo sapiens family with sequence similarity 110, member B (FAM110B), mRNA [NM_147189] |
| 325 | TREM2 | **0.475** | **0.001** | NM_018965 | ref\|Homo sapiens triggering receptor expressed on myeloid cells 2 (TREM2), transcript variant 1, mRNA [NM_018965] |
| 326 | PLK2 | **0.471** | **0.000** | NM_006622 | ref\|Homo sapiens polo-like kinase 2 (PLK2), transcript variant 1, mRNA [NM_006622] |
| 327 | ABCA3 | **0.469** | **0.001** | NM_001089 | ref\|Homo sapiens ATP-binding cassette, sub-family A (ABC1), member 3 (ABCA3), mRNA [NM_001089] |
| 328 | ANKRD35 | **0.465** | **0.002** | NM_144698 | ref\|Homo sapiens ankyrin repeat domain 35 (ANKRD35), transcript variant 1, mRNA [NM_144698] |
| 329 | PIK3R5 | **0.463** | **0.010** | NM_014308 | ref\|Homo sapiens phosphoinositide-3-kinase, regulatory subunit 5 (PIK3R5), transcript variant 2, mRNA [NM_014308] |
| 330 | TIMP3 | **0.462** | **0.000** | NM_000362 | ref\|Homo sapiens TIMP metallopeptidase inhibitor 3 (TIMP3), mRNA [NM_000362] |
| 331 | PI15 | **0.460** | **0.001** | NM_015886 | ref\|Homo sapiens peptidase inhibitor 15 (PI15), mRNA [NM_015886] |
| 332 | PTGS2 | **0.459** | **0.004** | NM_000963 | ref\|Homo sapiens prostaglandin-endoperoxide synthase 2 (prostaglandin G/H synthase and cyclooxygenase) (PTGS2), mRNA [NM_000963] |
| 333 | DBP | **0.458** | **0.001** | NM_001352 | ref\|Homo sapiens D site of albumin promoter (albumin D-box) binding protein (DBP), mRNA [NM_001352] |
| 334 | KIAA1462 | **0.457** | **0.000** | NM_020848 | ref\|Homo sapiens KIAA1462 (KIAA1462), mRNA [NM_020848] |
| 335 | CA12 | **0.455** | **0.001** | NM_001218 | ref\|Homo sapiens carbonic anhydrase XII (CA12), transcript variant 1, mRNA [NM_001218] |
| 336 | EDNRA | **0.454** | **0.002** | NM_001957 | ref\|Homo sapiens endothelin receptor type A (EDNRA), transcript variant 1, mRNA [NM_001957] |
| 337 | AK002210 | **0.450** | **0.024** | AK002210 | gb\|Homo sapiens cDNA FLJ11348 fis, clone PLACE4000638. [AK002210] |
| 338 | ADAMTS5 | **0.444** | **0.001** | NM_007038 | ref\|Homo sapiens ADAM metallopeptidase with thrombospondin type 1 motif, 5 (ADAMTS5), mRNA [NM_007038] |
| 339 | TMEM38A | **0.444** | **0.001** | NM_024074 | ref\|Homo sapiens transmembrane protein 38A (TMEM38A), mRNA [NM_024074] |
| 340 | HS6ST3 | **0.443** | **0.035** | NM_153456 | ref\|Homo sapiens heparan sulfate 6-O-sulfotransferase 3 (HS6ST3), mRNA [NM_153456] |
| 341 | TNFRSF11B | **0.442** | **0.000** | NM_002546 | ref\|Homo sapiens tumor necrosis factor receptor superfamily, member 11b (TNFRSF11B), mRNA [NM_002546] |
| 342 | TXNIP | **0.438** | **0.000** | NM_006472 | ref\|Homo sapiens thioredoxin interacting protein (TXNIP), mRNA [NM_006472] |
| 343 | EXOC3L2 | **0.436** | **0.031** | NM_138568 | ref\|Homo sapiens exocyst complex component 3-like 2 (EXOC3L2), mRNA [NM_138568] |
| 344 | CEACAM1 | **0.436** | **0.047** | NM_001712 | ref\|Homo sapiens carcinoembryonic antigen-related cell adhesion molecule 1 (biliary glycoprotein) (CEACAM1), transcript variant 1, mRNA [NM_001712] |
| 345 | RANBP3L | **0.434** | **0.000** | NM_001161429 | ref\|Homo sapiens RAN binding protein 3-like (RANBP3L), transcript variant 1, mRNA [NM_001161429] |
| 346 | ADRB2 | **0.432** | **0.001** | NM_000024 | ref\|Homo sapiens adrenoceptor beta 2, surface (ADRB2), mRNA [NM_000024] |
| 347 | HS6ST1 | **0.426** | **0.000** | NM_004807 | ref\|Homo sapiens heparan sulfate 6-O-sulfotransferase 1 (HS6ST1), mRNA [NM_004807] |
| 348 | FA2H | **0.425** | **0.001** | NM_024306 | ref\|Homo sapiens fatty acid 2-hydroxylase (FA2H), mRNA [NM_024306] |
| 349 | NPAS3 | **0.423** | **0.000** | NM_022123 | ref\|Homo sapiens neuronal PAS domain protein 3 (NPAS3), transcript variant 2, mRNA [NM_022123] |
| 350 | RAB17 | **0.421** | **0.000** | NM_022449 | ref\|Homo sapiens RAB17, member RAS oncogene family (RAB17), transcript variant 1, mRNA [NM_022449] |
| 351 | NOG | **0.415** | **0.004** | NM_005450 | ref\|Homo sapiens noggin (NOG), mRNA [NM_005450] |
| 352 | LMO2 | **0.410** | **0.000** | NM_005574 | ref\|Homo sapiens LIM domain only 2 (rhombotin-like 1) (LMO2), transcript variant 1, mRNA [NM_005574] |
| 353 | PAX9 | **0.409** | **0.000** | NM_006194 | ref\|Homo sapiens paired box 9 (PAX9), mRNA [NM_006194] |
| 354 | FOXA1 | **0.407** | **0.004** | NM_004496 | ref\|Homo sapiens forkhead box A1 (FOXA1), mRNA [NM_004496] |
| 355 | KCNE4 | **0.407** | **0.001** | NM_080671 | ref\|Homo sapiens potassium channel, voltage gated subfamily E regulatory beta subunit 4 (KCNE4), mRNA [NM_080671] |
| 356 | PARD6B | **0.405** | **0.000** | NM_032521 | ref\|Homo sapiens par-6 family cell polarity regulator beta (PARD6B), mRNA [NM_032521] |
| 357 | SIPA1L2 | **0.404** | **0.000** | NM_020808 | ref\|Homo sapiens signal-induced proliferation-associated 1 like 2 (SIPA1L2), mRNA [NM_020808] |
| 358 | TMEM132B | **0.402** | **0.000** | NM_052907 | ref\|Homo sapiens transmembrane protein 132B (TMEM132B), transcript variant 1, mRNA [NM_052907] |
| 359 | NFE2 | **0.399** | **0.000** | NM_006163 | ref\|Homo sapiens nuclear factor, erythroid 2 (NFE2), transcript variant 1, mRNA [NM_006163] |
| 360 | CLEC1A | **0.396** | **0.001** | NM_001297750 | ref\|Homo sapiens C-type lectin domain family 1, member A (CLEC1A), transcript variant 4, mRNA [NM_001297750] |
| 361 | BHLHE22 | **0.394** | **0.003** | NM_152414 | ref\|Homo sapiens basic helix-loop-helix family, member e22 (BHLHE22), mRNA [NM_152414] |
| 362 | TGFBR3 | **0.390** | **0.000** | NM_003243 | ref\|Homo sapiens transforming growth factor, beta receptor III (TGFBR3), transcript variant 1, mRNA [NM_003243] |
| 363 | PRM2 | **0.390** | **0.020** | NM_001286359 | ref\|Homo sapiens protamine 2 (PRM2), transcript variant 5, mRNA [NM_001286359] |
| 364 | NSG1 | **0.389** | **0.004** | NM_001287763 | ref\|Homo sapiens neuron specific gene family member 1 (NSG1), transcript variant 3, mRNA [NM_001287763] |
| 365 | ENTPD8 | **0.388** | **0.003** | NM_001033113 | ref\|Homo sapiens ectonucleoside triphosphate diphosphohydrolase 8 (ENTPD8), transcript variant 1, mRNA [NM_001033113] |
| 366 | CD86 | **0.365** | **0.046** | NM_006889 | ref\|Homo sapiens CD86 molecule (CD86), transcript variant 2, mRNA [NM_006889] |
| 367 | LRRTM4 | **0.365** | **0.011** | NM_024993 | ref\|Homo sapiens leucine rich repeat transmembrane neuronal 4 (LRRTM4), transcript variant 2, mRNA [NM_024993] |
| 368 | RNF43 | **0.363** | **0.014** | NM_017763 | ref\|Homo sapiens ring finger protein 43 (RNF43), mRNA [NM_017763] |
| 369 | WDR86 | **0.357** | **0.004** | NM_001284260 | ref\|Homo sapiens WD repeat domain 86 (WDR86), transcript variant 1, mRNA [NM_001284260] |
| 370 | ETNPPL | **0.347** | **0.010** | NM_031279 | ref\|Homo sapiens ethanolamine-phosphate phospho-lyase (ETNPPL), transcript variant 1, mRNA [NM_031279] |
| 371 | APCDD1 | **0.344** | **0.000** | NM_153000 | ref\|Homo sapiens adenomatosis polyposis coli down-regulated 1 (APCDD1), mRNA [NM_153000] |
| 372 | SLC4A4 | **0.342** | **0.024** | NM_003759 | ref\|Homo sapiens solute carrier family 4 (sodium bicarbonate cotransporter), member 4 (SLC4A4), transcript variant 2, mRNA [NM_003759] |
| 373 | FAM65C | **0.342** | **0.000** | NM_001290268 | ref\|Homo sapiens family with sequence similarity 65, member C (FAM65C), transcript variant 2, mRNA [NM_001290268] |
| 374 | CXCL3 | **0.340** | **0.003** | NM_002090 | ref\|Homo sapiens chemokine (C-X-C motif) ligand 3 (CXCL3), mRNA [NM_002090] |
| 375 | CD22 | **0.336** | **0.021** | NM_001771 | ref\|Homo sapiens CD22 molecule (CD22), transcript variant 1, mRNA [NM_001771] |
| 376 | CABYR | **0.323** | **0.000** | NM_012189 | ref\|Homo sapiens calcium binding tyrosine-(Y)-phosphorylation regulated (CABYR), transcript variant 1, mRNA [NM_012189] |
| 377 | FAM91A1 | **0.322** | **0.001** | NM_144963 | ref\|Homo sapiens family with sequence similarity 91, member A1 (FAM91A1), mRNA [NM_144963] |
| 378 | PAPPA | **0.317** | **0.000** | NM_002581 | ref\|Homo sapiens pregnancy-associated plasma protein A, pappalysin 1 (PAPPA), mRNA [NM_002581] |
| 379 | NXPH3 | **0.313** | **0.000** | NM_007225 | ref\|Homo sapiens neurexophilin 3 (NXPH3), mRNA [NM_007225] |
| 380 | BMP1 | **0.312** | **0.000** | ENST00000471755 | ens\|bone morphogenetic protein 1 [Source:HGNC Symbol;Acc:HGNC:1067] [ENST00000471755] |
| 381 | SLCO4A1 | **0.312** | **0.001** | NM_016354 | ref\|Homo sapiens solute carrier organic anion transporter family, member 4A1 (SLCO4A1), mRNA [NM_016354] |
| 382 | ADH1A | **0.310** | **0.009** | NM_000667 | ref\|Homo sapiens alcohol dehydrogenase 1A (class I), alpha polypeptide (ADH1A), mRNA [NM_000667] |
| 383 | TMEM100 | **0.291** | **0.000** | NM_018286 | ref\|Homo sapiens transmembrane protein 100 (TMEM100), transcript variant 2, mRNA [NM_018286] |
| 384 | RASL11A | **0.290** | **0.001** | NM_206827 | ref\|Homo sapiens RAS-like, family 11, member A (RASL11A), mRNA [NM_206827] |
| 385 | CFTR | **0.263** | **0.000** | NM_000492 | ref\|Homo sapiens cystic fibrosis transmembrane conductance regulator (ATP-binding cassette sub-family C, member 7) (CFTR), mRNA [NM_000492] |
| 386 | KLRC4 | **0.262** | **0.009** | NM_013431 | ref\|Homo sapiens killer cell lectin-like receptor subfamily C, member 4 (KLRC4), mRNA [NM_013431] |
| 387 | SUSD4 | **0.257** | **0.006** | ENST00000342943 | ens\|sushi domain containing 4 [Source:HGNC Symbol;Acc:HGNC:25470] [ENST00000342943] |
| 388 | PTGS1 | **0.253** | **0.000** | NM_000962 | ref\|Homo sapiens prostaglandin-endoperoxide synthase 1 (prostaglandin G/H synthase and cyclooxygenase) (PTGS1), transcript variant 1, mRNA [NM_000962] |
| 389 | HCAR3 | **0.248** | **0.009** | NM_006018 | ref\|Homo sapiens hydroxycarboxylic acid receptor 3 (HCAR3), mRNA [NM_006018] |
| 390 | FGG | **0.247** | **0.000** | NM_000509 | ref\|Homo sapiens fibrinogen gamma chain (FGG), transcript variant gamma-A, mRNA [NM_000509] |
| 391 | PODXL | **0.244** | **0.000** | NM_001018111 | ref\|Homo sapiens podocalyxin-like (PODXL), transcript variant 1, mRNA [NM_001018111] |
| 392 | CBLN2 | **0.234** | **0.000** | NM_182511 | ref\|Homo sapiens cerebellin 2 precursor (CBLN2), mRNA [NM_182511] |
| 393 | KIT | **0.227** | **0.034** | NM_000222 | ref\|Homo sapiens v-kit Hardy-Zuckerman 4 feline sarcoma viral oncogene homolog (KIT), transcript variant 1, mRNA [NM_000222] |
| 394 | GPR182 | **0.219** | **0.043** | NM_007264 | ref\|Homo sapiens G protein-coupled receptor 182 (GPR182), mRNA [NM_007264] |
| 395 | ADH1C | **0.198** | **0.000** | NM_000669 | ref\|Homo sapiens alcohol dehydrogenase 1C (class I), gamma polypeptide (ADH1C), mRNA [NM_000669] |
| 396 | CHRM1 | **0.198** | **0.000** | NM_000738 | ref\|Homo sapiens cholinergic receptor, muscarinic 1 (CHRM1), mRNA [NM_000738] |
| 397 | PTPLB | **0.198** | **0.000** | NM_198402 | ref\|Homo sapiens protein tyrosine phosphatase-like (proline instead of catalytic arginine), member b (PTPLB), mRNA [NM_198402] |
